# Deciphering the liquid–liquid phase separation induced modulation in the structure, dynamics, and enzymatic activity of an ordered protein β-lactoglobulin†

**DOI:** 10.1039/d3sc06802a

**Published:** 2024-02-08

**Authors:** Saurabh Rai, Srikrishna Pramanik, Saptarshi Mukherjee

**Affiliations:** a Department of Chemistry, Indian Institute of Science Education and Research Bhopal Bhopal Bypass Road Bhopal 462066 Madhya Pradesh India saptarshi@iiserb.ac.in

## Abstract

Owing to the significant role in the subcellular organization of biomolecules, physiology, and the realm of biomimetic materials, studies related to biomolecular condensates formed through liquid–liquid phase separation (LLPS) have emerged as a growing area of research. Despite valuable contributions of prior research, there is untapped potential in exploring the influence of phase separation on the conformational dynamics and enzymatic activities of native proteins. Herein, we investigate the LLPS of β-lactoglobulin (β-LG), a non-intrinsically disordered protein, under crowded conditions. In-depth characterization through spectroscopic and microscopic techniques revealed the formation of dynamic liquid-like droplets, distinct from protein aggregates, driven by hydrophobic interactions. Our analyses revealed that phase separation can alter structural flexibility and photophysical properties. Importantly, the phase-separated β-LG exhibited efficient enzymatic activity as an esterase; a characteristic seemingly exclusive to β-LG droplets. The droplets acted as robust catalytic crucibles, providing an ideal environment for efficient ester hydrolysis. Further investigation into the catalytic mechanism suggested the involvement of specific amino acid residues, rather than general acid or base catalysis. Also, the alteration in conformational distribution caused by phase separation unveils the latent functionality. Our study delineates the understanding of protein phase separation and insights into the diverse catalytic strategies employed by proteins. It opens exciting possibilities for designing functional artificial compartments based on phase-separated biomolecules.

## Abstract 2

Liquid–liquid phase separation (LLPS) exhibited by an ordered protein β-lactoglobulin (β-LG) under crowded environment prompts alterations in conformational distribution leading to emergence of new promiscuous (esterase-like) activity.

## Graphical abstract

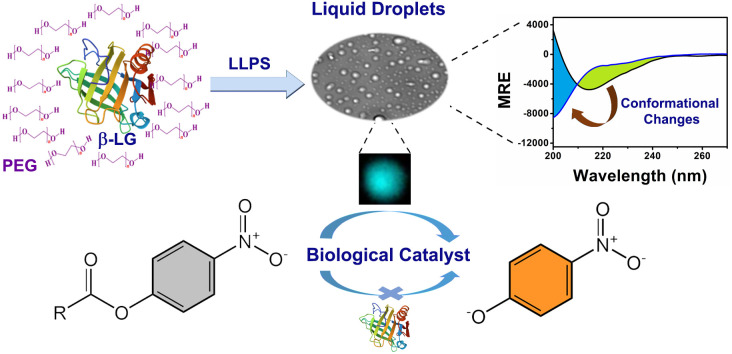

## 1 Introduction

**Fig. 1:**
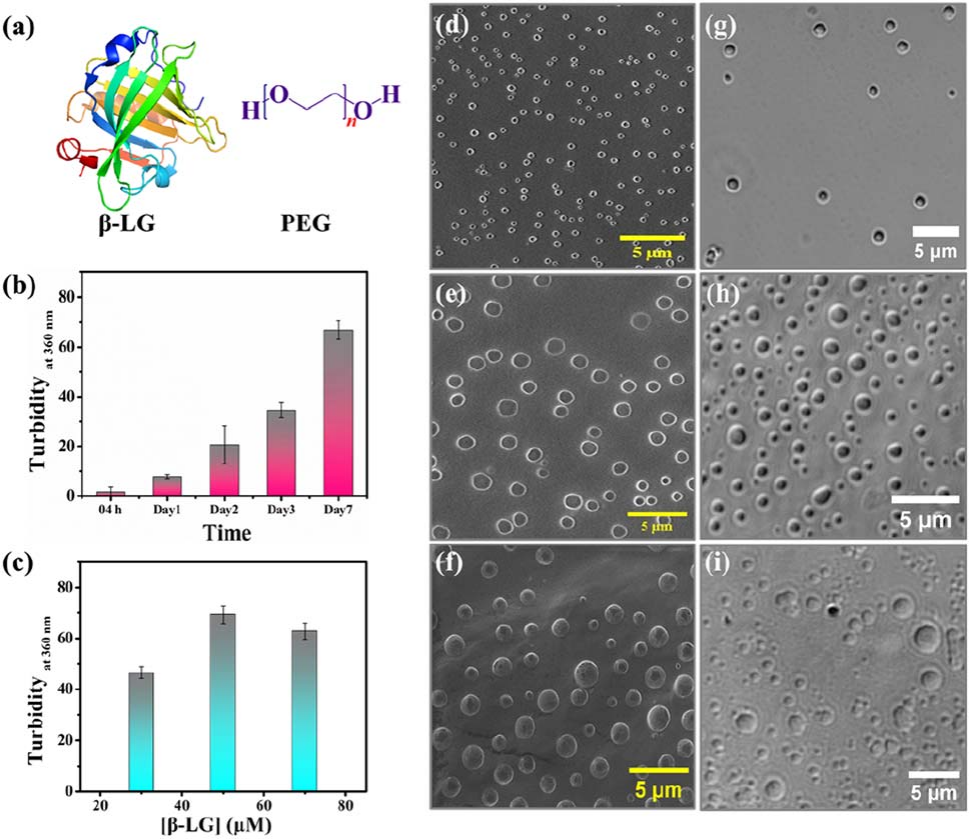
Experimental investigation of phase separation conditions for β-lactoglobulin (β-LG) in the presence of polyethylene glycol (PEG8000) at 37 °C and pH 7.4. (a) Molecular structures of β-LG and PEG (*n* = 8000). (b) Time-dependent turbidity analysis of 50 μM β-LG incubated with 10% w/v PEG8000 at 37 °C and pH 7.4. (c) Turbidity profiles of β-LG incubated with a fixed concentration of 10% w/v PEG8000 at different protein concentrations at 37 °C and pH 7.4. (d)–(f) Morphological evolution of droplets observed through FESEM studies during incubation of 50 μM β-LG with 10% w/v PEG8000 at 37 °C and pH 7.4 for 1, 3 and 7 day(s), respectively. (g)–(i) Corresponding DIC images of 50 μM β-LG incubated with 10% w/v PEG8000 at 37 °C and pH 7.4 for 1, 3 and 7 day(s), respectively.

Bimolecular condensates formed through liquid–liquid phase separation (LLPS) of proteins, polypeptides, and nucleic acids have garnered significant research interest due to their profound impact on subcellular organization, physiology, and the realm of biomimetic materials.^1–5^ Recent investigations reveal that intracellular biomolecule-rich phase-separated systems are formed through multivalent macromolecular interactions such as electrostatic, π–π, hydrogen bonding, and hydrophobic interactions.^6–8^ The behavior of the phase separation process, depending on the local molecular environment and density is fundamental in regulating several biological processes and also creates heterogeneous phases inside cells forming membraneless organelles (MLO).^9–12^ Such an environment inside a MLO not only modifies the functional properties of these biomolecules but also induces several conformational transitions.^13^ The LLPS phenomenon has been demonstrated to be the main process in protein assembly, and the crystallization of the globular protein, human serum albumin.^14,15^ Most importantly, liquid-like droplets formed *via* phase separation processes may consist of an aggregated state of proteins that are often associated with the onset of neurodegenerative diseases.^16–18^ Most of the studies on the phase-separating system have explored the intrinsically disordered regions of the protein that undergo a structural transition from random coil to the aggregated state; the aggregation behavior being irreversible.^19,20^ However, more in-depth characterization of globular proteins undergoing LLPS,^21,22^ and elucidation of their conformational and functional behaviors in such a small-volume droplet-like phase are needed to decipher the molecular mechanism underlying the formation of MLOs and to develop novel functional bionanomaterials and carriers in drug therapies to prevent disease-related condensates. Herein, we delve into understanding the consequences of the phase separation process for a representative globular protein β-lactoglobulin (β-LG), which has not been reported to have any intrinsic disorders or low complexity domains (LCD), typical features of proteins undergoing LLPS.^21^ In particular, we aimed to comprehend the conformational dynamics of β-LG in the phase-separated state to determine how its activities can be finetuned for the development of biomaterials for catalytic activity. β-LG is a globular protein containing 162 amino acid residues with a β-sheet-rich secondary structure (Fig. 1a) and is well-known for its ability to bind and transport a wide variety of insoluble bioactive components.^23–25^ Additionally, it has been reported that it can enhance or modulate human immune responses.^26^ Herein, we have used polyethylene glycol, PEG8000 (Fig. 1a) as an inert crowder that can mimic a cellular-like macromolecular crowding environment. PEG has been employed to achieve an aqueous two-phase system for separating and purifying α-LG and β-LG from the concentrated whey proteins.^27^ The local and global conformations, and concentrations of biomolecules, like nucleic acids and proteins, can be vividly modulated in a crowded environment created by an inert crowder, like PEG8000.^28–30^ Very recently, it has been found that different ordered and disordered proteins can undergo LLPS in the presence of a crowder, like PEG.^21^ The formation of the phase-separated liquid droplets occurred through non-covalent interactions between the proteins, and such droplets were characterized using various spectroscopic and microscopic techniques. The fluid-like behavior and dynamics of these droplets were characterized by fluorescence recovery after photobleaching (FRAP), and time-resolved rotational anisotropy studies using the fluorescently tagged protein.

Most interestingly, our study revealed that the condensation of β-LG within droplets can significantly boost enzymatic activity in the hydrolysis of various chromogenic and fluorogenic esterase substrates. It is important to clarify that such modulations in the catalytic activity were neither observed in the native, denatured, or fibrillar forms of β-LG, nor other proteins like HSA and BSA. This is the first-ever systematic report that sheds light on the conformational and catalytic activity of an ordered protein after the formation of phase-separated liquid droplets. Circular dichroism (CD) spectroscopy revealed that β-LG undergoes a structural transition from a predominantly β-sheet form to an α-helical form upon the treatment with 2,2,2-trifluoroethanol (TFE, discussed later). This work proposes a new insight for the development of phase-separated biomolecule-based catalytic coacervates.

## 2 Experimental section

### 2.1 Materials

β-Lactoglobulin (β-LG), polyethylene glycol with an average molecular weight of 8000 g mol^−1^ (PEG8000), guanidine hydrochloride (GdHCl), 1,6-hexanediol, 2,2,2-trifluoroethanol (TFE), thioflavin T (ThT), sodium chloride (NaCl), sodium dihydrogen phosphate monohydrate (NaH_2_PO_4_·H_2_O), disodium hydrogen phosphate heptahydrate (Na_2_HPO_4_·7H_2_O), *p*-nitrophenyl acetate (PNPA), *p*-nitrophenyl butyrate (PNPB), *p*-nitrophenyl valerate (PNPV), 2′,7′-dichlorofluorescein diacetate (DCFDA), caproic acid (hexanoic acid), deuterium oxide (D_2_O), dextran and sodium acetate trihydrate (CH_3_COONa·3H_2_O) were used as received from Sigma-Aldrich, U.S.A. β-LG (>90%) contains genetic variants β-lactoglobulins A and B (β-LG A and β-LG B). Dyes 7-(diethylamino)-3-(4-maleimidylphenyl)-4-methyl coumarin (CPM), 5-([4,6-dichlorotriazin-2-yl]amino) fluorescein hydrochloride (5-DTAF) and rhodamine 6G (Rh6G) were all purchased from Sigma-Aldrich, U.S.A. All the chemicals were of the highest grade and were used without any further purification. Phosphate buffer (10 mM, pH 7.4) solutions were prepared using deionized Milli-Q water. Milli-Q water was obtained from a Millipore water purifier system (Milli-Q integral). The concentration of β-LG was 5 μM for CD spectroscopic measurements. For all other characterization studies, the concentration of β-LG was 50 μM.

### 2.2 Methods

#### 2.2.1 Steady-state measurements

For steady-state absorption measurements, a Cary-100 UV-vis spectrophotometer was used in the scanning range of 200 nm to 800 nm. A HORIBA JOBIN YVON, FLUOROLOG 3-111 fluorimeter was used for steady-state fluorescence measurements in a 1 cm path length quartz cuvette with an integration time of 0.1 s. Both the excitation and the emission slits were kept equal (2 nm each for intrinsic fluorescence, 1/2 nm each for external fluorophores) and the room temperature was maintained at 23 °C for all the measurements. The final protein concentration was kept at 50 μM for all of the fluorescence studies. The samples were excited using a 450-Watt Xenon arc lamp at various required wavelengths (295 nm for intrinsic tryptophan, 320 nm for the charge transfer (CT), 384 nm for CPM labeled β-LG, and 530 nm for Rh6G), and emission signals were collected in the appropriate range for spectral acquisition.

#### 2.2.2 Time-resolved fluorescence anisotropy

For time-resolved anisotropy experiments, samples were excited using IBH-NanoLED sources, a picosecond diode (*λ*_ex_: 295 nm for intrinsic fluorescence measurements, and 405 nm for the CPM-labeled β-LG). The details of the experimental setup are mentioned elsewhere.^31^ The instrument response function (IRF) was recorded using an aqueous solution of Ludox (800 ps for 295 nm source, and 108 ps for 405 nm source). The goodness of the fitted data was evaluated by the fitting parameters (*χ*^2^) as well as the visual assessment of the residuals. For the anisotropy measurements, the tryptophan fluorescence sample was excited using a 295 nm diode laser, while collecting the decay at 340 nm. For CPM labeled β-LG, the sample was excited using a 405 nm diode laser, while collecting the decay at 470 nm. The fluorescence anisotropy (*r*(*t*)) was calculated using the following equation:^31^1
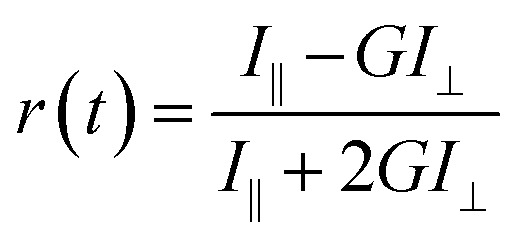
*I*_‖_ and *I*_⊥_ respectively, denote the parallel and perpendicular fluorescence decay intensities and *G* is the instrumental factor.

#### 2.2.3 Turbidity assay

For turbidity measurement of solutions, UV-visible absorbance spectra were recorded at 360 nm on a spectrophotometer (Cary 100 UV-vis spectrophotometer) which were further processed to obtain turbidity of the sample using the following equation:^22^

2 *T* = 100 − (100 × 10^−*A*^)

Here, the abbreviation *T* stands for turbidity, and *A* stands for absorbance.

#### 2.2.4 ThT fluorescence assay

From a concentrated stock solution (1 mM) of ThT, 4 μL was added to the sample solution to make a final concentration of 4 μM in 1 mL of solution. The protein concentration was maintained at 50 μM for all the measurements of the ThT fluorescence assay. All spectra were collected at *λ*_ex/em_ = 450/484 nm, after 5 min of incubation of ThT with the samples.

#### 2.2.5 Field emission scanning electron microscopy (FESEM)

FESEM images were obtained using the samples coated on a mica foil by a drop-casting technique which was attached to SEM stubs. The samples were dried overnight in a vacuum. Before FESEM imaging, the sample surface was coated with gold for 120 s. A Carl Zeiss (Ultra plus) FESEM instrument was used in our experiment to capture the SEM images at an accelerating voltage of 10–20 kV. For visualizing the coalescence, a concentrated solution was used.

#### 2.2.6 Confocal microscopy

Fluorescence lifetime imaging microscopy (FLIM) and fluorescence intensity-based images for β-LG droplets were recorded, respectively, on an inverted confocal microscope setup (PicoQuant, MicroTime 200, Olympus IX-71) and Olympus FV3000 confocal microscope as described elsewhere.^32–34^ For FLIM/fluorescence imaging, the liquid droplets were obtained by mixing 5% CPM-labeled proteins with unlabeled proteins to avoid any possible effect of CPM on the LLPS processes. For sample preparation, 5 μL of the phase-separated sample was drop cast on a clean microscope glass slide and covered with a commercially available coverslip. Commercial nail paint was used to seal the coverslip to stabilize and prevent solvent evaporation during the experiment. The sample was excited using a 405 nm excitation laser. Also, Rh6G dye was used to equilibrate with the phase-separated solution which was excited using laser excitation of 532 nm. For the FLIM experiments, we used a water immersion objective (magnification 60×, numerical aperture (NA) = 1.2) along with a dichroic mirror (HQ470/530 DCXR, Chroma) and a band-pass filter (HQ532lp, Chroma) to avoid the contribution of the excitation light to the emission signal. The emission was focused through a 50 μm pinhole and the emission signal was collected on a microphoton device detector (PicoQuant) and processed through a PicoHarp-300. For acquiring fluorescence intensity-based images, an oil immersion objective (a plain apochromatic 63X, 1.4 NA oil immersion objective) was used and the emission was collected through a DAPI channel for CPM-labelled samples. For further analysis of droplet images, ImageJ software was used.^34^

#### 2.2.7 Fluorescence recovery after photobleaching (FRAP)

FRAP experiments were carried out on an Olympus FV3000 confocal microscope using a FRAP module. The samples were observed with a plain apochromatic 63X, 1.4 NA oil immersion objective using a 405 nm laser. ∼5% CPM-labelled protein and 95% unlabeled protein were used to incubate 50 μM β-LG in 10% w/v PEG8000, at 37 °C and pH 7.4 for carrying out FRAP experiments. Bleaching was performed at 50% laser power during fly forward by ROI scan feature. Photobleaching was performed on a spherical region of interest (ROI) inside the droplet and subsequently, images were acquired every 1.6 s. The data were collected from 11 different independent experiments choosing different droplets and the ROIs. To analyze the fluorescence recovery, the background subtraction and photobleaching corrections were applied. The fluorescence recovery was analyzed using Cell Sens software (Olympus), where the half-lifetime (*τ*_1/2_) was determined as the time at which 50% fluorescence recovery occurred. The following double exponential fit equation was used for analyzing the recovery curves:^34^3
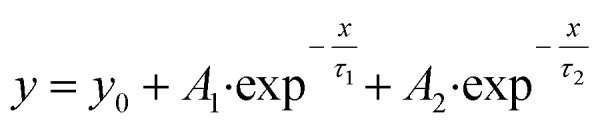


The average and standard deviation (s.d.) of the independent data (*N* = 11) were calculated and plotted using OriginPro 8.5 software.

#### 2.2.8 Rh6G incubation with liquid condensates

For this study, we incubated 1 μM of Rh6G with the liquid condensate solution of β-LG (50 μM incubated with 10% w/v PEG8000 at pH 7.4 and temperature 37 °C) for 12 h. The sample was imaged by using an excitation laser of 532 nm.

#### 2.2.9 Circular dichroism (CD) spectroscopy

For the conformational analysis, CD measurements were performed on a JASCO J-815 spectropolarimeter. All the CD measurements were performed with a 0.1 cm path length quartz cell at a temperature of 298 K (25 °C) and spectra were recorded by diluting both native and phase-separated systems (to a final concentration of 5 μM unless otherwise mentioned), keeping intensity within the instrument limitation to avoid the saturation of HT voltage. The scan speed was kept at 100 nm min^−1^ with proper baseline correction against the buffer signal. The average of three successive scans was taken for each spectrum, and the spectra were plotted using OriginPro 8.5 software. The results are expressed as mean residue ellipticity (MRE) in units of deg cm^2^ dmol^−1^, as follows:^32^4
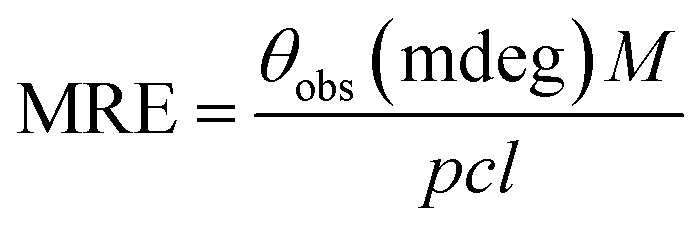
Here, *θ*_obs_ is the observed ellipticity in milli-degrees, *M* is the molecular weight of the protein in g dmol^−1^, *p* represents the number of amino acid residues, *l* is the path length of the cuvette, and *c* is the protein concentration in g L^−1^.

#### 2.2.10 Fluorescence labeling of β-LG with CPM dye

β-LG contains 5 cysteine residues with two disulfide bonds and one free cysteine residue in its native state. For the CPM labeling, free cysteine was utilized, and labeling was done according to the previously reported protocol with some modifications.^35^ In a 5 mL β-LG solution prepared in 50 mM phosphate buffer having pH 7.4, 5 μL of 69 mM CPM solution in DMSO was added to maintain a β-LG : CPM molar ratio of 1 : 2. The solution was stirred gently at 300 rpm at 25 °C. The unreacted dye was removed through dialysis using a 14 kDa cutoff dialysis tube in 10 mM PB at 4 °C for 36 h.

#### 2.2.11 Fluorescence labeling of PEG with 5-DTAF dye

The labelling of PEG was done according to the previously reported protocol.^36,37^ In brief, 1.4 mg of 5-DTAF was added to a 2 mL aqueous PEG8000 (2% w/v) solution followed by a dropwise addition of 600 μL 1% w/v Na_2_SO_4_ solution. The solution pH was adjusted to 9–10 with NaOH. The reaction proceeded for 3 h at 60 °C with continuous stirring in the dark. Subsequently, the polymer was precipitated using cold ethanol. The precipitate was thoroughly washed with ethanol and dried using a freeze dryer.

#### 2.2.12 Liquid–liquid phase separation of β-LG

LLPS of β-LG was observed upon incubation of different concentrations of the protein (20–100 μM) in phosphate buffer (PB, with or without NaCl) with polyethylene glycol (PEG8000) at pH 7.4 and temperature 37 °C. To conduct the pH-dependent study, acetate buffer (pH ∼2.3) was used to maintain a low pH. The samples in micro-centrifuge tubes were incubated in a solid-state temperature bath. Phase separation of β-LG was also monitored at different pH and temperatures that resulted in different extents of droplet formation. No droplet formation was observed at 4 °C, and high temperature (*viz*. 50 °C and above) suggests the upper critical solution transition behavior of β-LG. Droplet formation was confirmed and monitored through turbidity assays, FESEM, confocal microscopic DIC, fluorescence intensity, and lifetime (FLIM)-based images.

#### 2.2.13 Fibrillation of β-LG

To obtain fibrillar structures, the proteins were heated beyond melting temperatures under varied conditions of pH. For β-LG, the previously reported procedure was adopted.^38^ In brief, 2% (w/v) β-LG was taken in water and incubated at 90 °C for 6 h in a dry bath. Thereafter, the solution was quenched with a stream of cold water and used for further studies.

#### 2.2.14 Catalytic activity of the phase-separated liquid droplets

For calculating the velocity constant, 300 μL of the condensate solution in 10 mM PB was taken in which varying concentrations of PNPA, PNPB, and PNPV were added. For the control experiments using PB, PEG8000, native β-LG, BSA, and HSA, all the experimental conditions were kept the same. Every time fresh stock solutions of the *p*-nitrophenyl esters were made in UV-grade methanol to keep the final volume of 5 μL to be added to the final solution before the experiments. The absorbance kinetics was monitored at 400 nm for *p*-nitro phenolate (PNP). The reaction rate was measured from the kinetics of all the concentrations by linear fitting for 5 minutes. The catalytic rate constant (*k*_cat_) and Michaelis constant (*K*_m_) were obtained from the nonlinear curve fitting of velocity against substrate concentration and Michaelis Menten enzyme kinetic analysis. For the qualitative analysis of enzymatic activity through fluorescence spectroscopy, a stock solution of DCFDA (initially prepared in DMSO) was added into 1 mL of PB, PEG8000, native β-LG, and phase-separated β-LG solution, making a final concentration of DCFDA as 10 μM. The fluorescence spectra were recorded for measuring the different extent of 2′,7′-dichlorofluorescein (DCF) produced at different time intervals up to 24 h by using an excitation wavelength of 490 nm, keeping both the excitation and emission slit widths of 1 nm. The absorbance kinetic studies were performed using a SHIMADZU UV-2600i UV-vis spectrophotometer.

#### 2.2.15 Electrospray ionization spectroscopy

The ESI-MS spectrum of the hydrolyzed product of PNPA was acquired on a Bruker microTOF QII high-resolution mass spectrometer. The sample was prepared by decanting the supernatant solution after centrifugation followed by filtration through a syringe filter having a pore size of 0.22 μm.

#### 2.2.16 Solvent isotopic effect and medium-chain fatty acid experiment

The deuterium oxide isotope effect was analyzed to decipher the role of proton transfer and the involvement of water molecules. The influence of D_2_O on the ester hydrolysis of PNPA by β-LG condensates was investigated to understand the behavior of the proton. For the solvent isotopic study, 10% w/v PEG8000 solution prepared in D_2_O and H_2_O, separately, were used to half dilute the condensate solution (prepared by incubating 50 μM β-LG solution at pH 7.4 and temperature 37 °C for 7 days). With these diluted solutions, PNPA hydrolysis kinetics experiments were conducted keeping all other conditions the same (using 120 μM PNPA and recording the absorbance at 400 nm). To investigate the effect of medium chain fatty acids, we added caproic acid to the condensate solution maintaining the final concentration of 0.0003% w/v and performed the PNPA hydrolysis kinetics (using 120 μM PNPA and recording the absorbance at 400 nm).

#### 2.2.17 Denaturation of β-LG by urea and GdHCl

50 μM β-LG was incubated with varying concentrations of urea (0–8 M) and GdHCl (0–6 M) in HCl/KCl buffer at pH ∼2.3 and 23 °C. For further investigations, the protein was diluted in such a way that the denaturant concentration remained the same. The presence of denaturants (GdHCl and urea) caused the HT value of the CD detector to shoot up beyond the permissible value for spectral acquisition below 210 nm. Hence, for the denatured protein, the spectra could not be recorded below 210 nm.

#### 2.2.18 Conformational alteration of β-LG by TFE

The required concentration of β-LG (10 μM for CD and 50 μM for PNPA kinetics) was incubated with varying concentrations of TFE (0–30% v/v) in the solution at different pH (2.3 and 7.4) and 23 °C. After 10 minutes, the solutions were examined through CD spectral measurements and used for further experiments.

## 3 Results and discussion

### 3.1 Characterization of LLPS of β-LG in a crowded environment

In the quest to find the phase separation conditions, we incubated β-LG with an inert synthetic crowder, PEG8000 at different temperatures, pH, and concentrations. The enhanced turbidity has been considered as the initial indicator for the phase separation. We systematically monitored the time-dependent turbidity of different concentrations of the protein solutions and noted the changes in the turbidity of β-LG, both as a function of time and concentration of the protein (Fig. 1b and c). Next, we proceeded with 50 μM protein to further optimize the role of pH, temperature, time, and PEG8000 concentration in the phase separation process of β-LG. From the turbidity assay (Fig. S1a–c†), it can be rationalized that β-LG undergoes time-dependent phase separation in a crowded environment around physiological conditions. This signifies the role of the microenvironment in inducing such MLO of proteins. To confirm whether this turbidity originates from the phenomenon of LLPS, we performed the Thioflavin T (ThT) assay where we observed no significant increase in the ThT fluorescence (which is typically seen when the protein gets aggregated) with the formation of β-LG condensates. However, as expected, the fluorescence recorded in the presence of aggregated protein was different and much more intense than what was observed in the case of liquid condensates (Fig. S2†). This study thus ruled out the possibility of fibrillation or amyloidogenic aggregation in the system when subjected to the molecular crowder, PEG8000, and other environmental factors that exclusively trigger the formation of liquid-like droplets.

We further investigated the phase separation process by employing field emission scanning electron microscopy (FESEM) and differential interference contrast (DIC) images, simultaneously. From the FESEM (Fig. 1d–f) as well as DIC images (Fig. 1g–i), we observed the formation of sphere-like droplets which represent the condensates of β-LG. The increased size of the droplets over time can be ascribed to the fusion/coalescence (Fig. S3a†), and the observed surface wettability (Fig. S3b†) indicates the liquid-like properties of these condensates. When β-LG was incubated in the absence of PEG8000 (Fig. S3c and d†), or PEG8000 was incubated without the protein (Fig. S3e and f†), no such spherical morphologies were observed. This substantiates the importance of PEG8000 for the formation of the droplets. As expected for the aggregated protein, FESEM and DIC images displayed a completely different morphology when compared to the liquid-like condensates (Fig. S4a and b†). This further supports the idea that the observed droplets are distinct from protein aggregates.

**Fig. 2:**
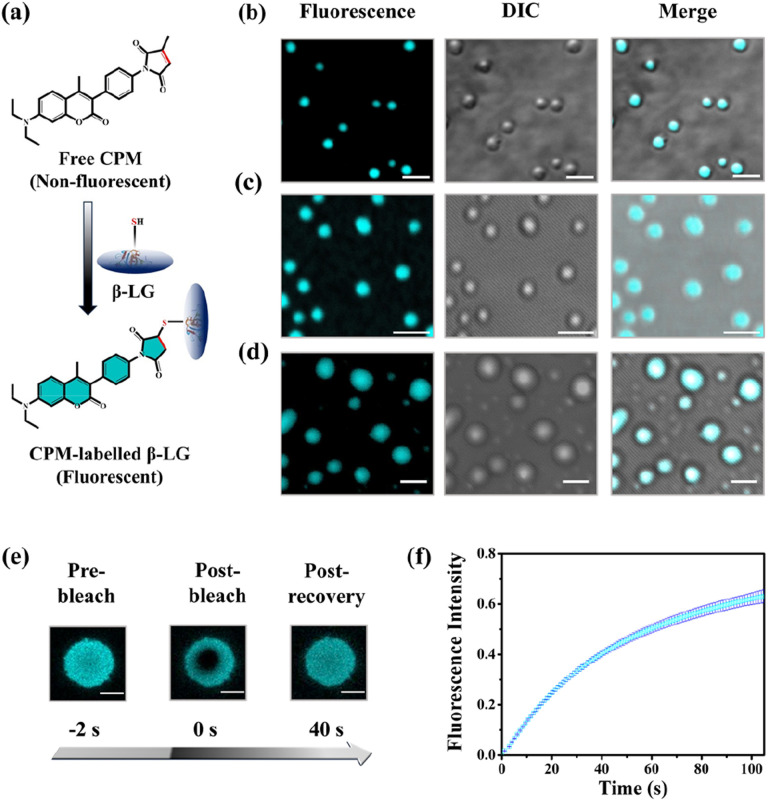
Characterization of the CPM-labelled β-LG droplets and FRAP. (a) Schematic representation of the labelling process of β-LG with CPM dye. (b)–(d) Represent confocal microscopy images (left to right: fluorescence intensity-based images, DIC images and the colocalization images) of the β-LG droplets (50 μM β-LG, with 5% CPM-labelled β-LG, in 10% w/v PEG8000, at 37 °C and pH 7.4) for day1, day3 and day7, respectively (scale bar: 3 μm). (e) Fluorescence images of β-LG droplets (50 μM β-LG, with 5% CPM-labelled, in 10% w/v PEG8000, at 37 °C and pH 7.4 for day5) (obtained by photobleaching a ROI) before bleaching, after bleaching, and post-recovery of fluorescence during the FRAP experiments (scale bar: 3 μm). (f) FRAP kinetics of phase-separated β-LG droplets (50 μM β-LG, with 5% CPM-labelled, in 10% w/v PEG8000, at 37 °C and pH 7.4), demonstrating rapid fluorescence recovery with an average half-lifetime of 60 seconds. The error bar represents mean ± standard deviation (s.d.) for *n* = 11 independent experiments.

To understand whether these liquid-like droplets were formed by proteins, we performed fluorescence confocal microscopy experiments. For this, we covalently labeled the protein (Fig. 2a) with 7-(diethylamino)-3-(4-maleimidylphenyl)-4-methyl coumarin (CPM) dye using the optimized protocol with slight modifications,^37^ and such labeling has minimum effect on the secondary structure of the proteins.^37^ The images of the condensate solution (containing 5% labeled protein along with unlabeled protein) show distinctly visible fluorescent droplets (Fig. 2b–d) well-matched with DIC images which were well observable for up to 7 days. This substantiates that these droplets are the condensates of the protein with a much higher local density in the droplet phase as compared to the surrounding solution. Additionally, the morphology of these droplets is completely different from what was observed for the aggregates (Fig. S4c†). To ensure that these droplets exhibit fluid-like properties and are not solid aggregates, we further characterized these droplets using the fluorescence recovery after photobleaching (FRAP) technique.^39^ In this experiment, we exposed a region of interest (ROI) in a droplet to the incident laser with 100% power for a short period of 2 s, and the process of fluorescence recovery was monitored through the subsequent image acquisition to obtain the FRAP kinetics curve. As seen in Fig. 2e, S5a and Video S1,† the fluorescence emission from the bleached droplets recovers gradually with time, resulting in an almost uniform distribution indicating the diffusion of protein chains in the liquid condensates. From the average of 11 independent experiments, we observed a recovery of 50% within 60 s (Fig. 2f and S5b†), which closely aligns with the recovery rates and half-life times typically observed for liquid condensates.^1,2^ On the other hand, it has been observed that proteins that exhibited a liquid-to-solid transition did not recover after photo-bleaching.^17^ This FRAP analysis thus attests to the liquid-like behaviour of these droplets, effectively eliminating the possibility of solid-like aggregates of protein molecules.

Moreover, rhodamine 6G (Rh6G) incubated with the protein-rich droplet solution revealed distinct fluorescent droplets with the partition of Rh6G within them (Fig. S5c†). These findings not only suggest the formation of membraneless architecture (resembling an MLO), but they also indicate the potential of these condensates to act as effective dye-trapping agents.

### 3.2 Molecular reorientation dynamics and conformational flexibility

**Fig. 3:**
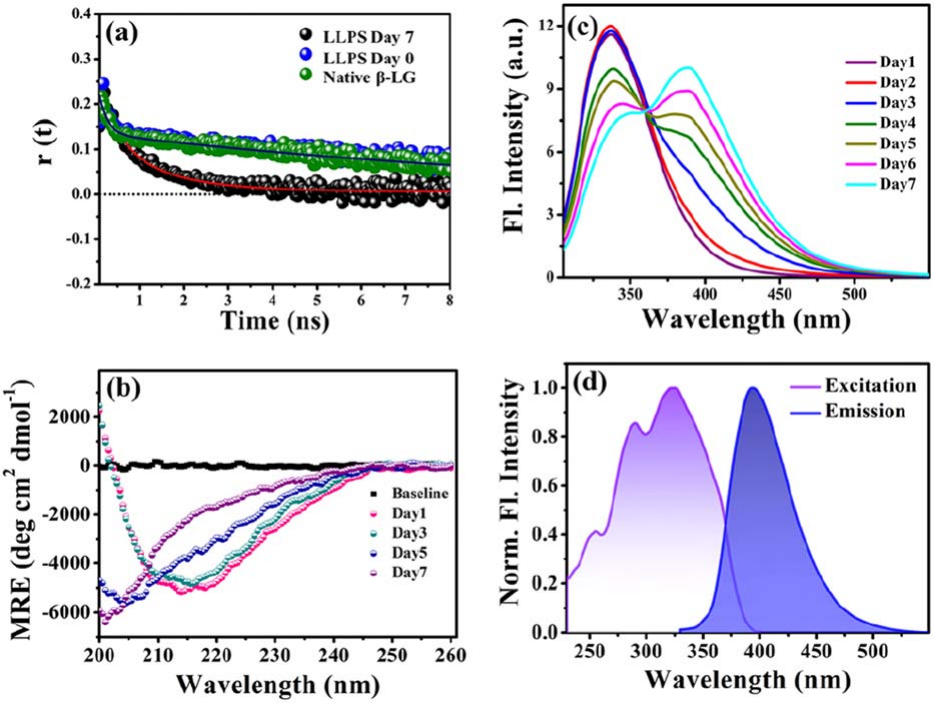
Characterizing protein conformation in the phase-separated state. (a) Anisotropy measurements using CPM-labelled protein for native and phase-separated states (50 μM β-LG, with 5% CPM-labelled β-LG, in 10% w/v PEG8000, at 37 °C and pH 7.4) reveal the dynamic molecular reorientation of the protein within the liquid condensates. (b) Far-UV CD spectra of 50 μM β-LG in 10% w/v PEG8000 (incubated at 37 °C and pH 7.4) recorded with a diluted solution at an effective protein concentration of 5 μM within the instrument limitation, to avoid the saturation of the detector. The dilution maintains the liquid droplet morphology unaffected (data not shown here). (c) The fluorescence spectra of β-LG undergoing the phase separation (*λ*_ex_ = 295 nm) display the gradual increase in charge transfer band intensity through FRET between the intrinsic tryptophan and the charge transfer species. (d) Charge transfer fluorescence excitation and emission spectra of the phase-separated state of β-LG centered around 320 nm and 400 nm, respectively.

To gain insights into the molecular reorientation dynamics of β-LG after the formation of condensates, time-resolved anisotropy measurements were conducted using both extrinsic and intrinsic fluorescence approaches. The fluorescence anisotropy decays of the native protein β-LG in an aqueous buffer solution and the phase-separated condensates under crowder milieu are depicted in Fig. 3a and S6,† and all the corresponding decay parameters are summarized in Table S1.† Notably, we observed a substantial decrement in the anisotropy (indicative of faster rotational time) for the phase-separated protein compared to the native protein. This finding suggests an extended conformation and increased mobility of the protein after phase separation, relative to its dispersed phase in the native state. The lower value of residual anisotropy for the phase-separated protein further attests to the augmented flexibility in this state as compared to the native state of the protein (Fig. S6†). Also, this change in anisotropy is not residue-specific, as a similar trend was observed for intrinsic rotor tryptophan (Fig. S6†) as well as the CPM labeled β-LG (Fig. 3a, Table S2†). Typically, in contrast to the aggregation of proteins, the phase-separated state of proteins is associated with rapid chain fluctuations, a red-shifted emission (discussed later), and a faster anisotropy.^40^ Therefore, these observations reaffirm the formation of liquid-like condensates of β-LG.

To understand the conformational changes of the protein inside these membrane-less compartments, we recorded circular dichroism (CD) spectra. For macromolecules like proteins, the dynamic secondary structures are characterized by signature peaks in CD spectra. Specifically, β-sheets are characterized by a large negative peak around 218 nm and a positive peak around 195 nm, while alpha-helices exhibit negative peaks around 208 nm and 222 nm.^41^ Random coil structures are characterized by low ellipticity above 210 nm and a span of spectrum with a negative band towards 200 nm. β-LG, a β-rich protein, possesses approximately 50% β-sheet content.^42^ As shown in Fig. 3b, the secondary structure of β-LG undergoes significant changes during the phase separation process. This change is evident from the decrease in β-sheet content as a consequence of phase separation, as indicated by the reduction in ellipticity magnitude around 218 nm. Notably, no substantial changes were observed when the phenomenon of phase separation was not operational, *i.e.*, when the protein was incubated without PEG8000 (Fig. S7†). This observation further substantiates the conclusion drawn from our time-resolved anisotropy measurements, reinforcing that phase separation leads to the more conformational flexibility of β-LG as compared to the compact β-sheet structure observed in its native state. To gain deeper insights into the conformational and rotational dynamics of the protein within the phase separation regime, we separated the condensed phase from the dilute phase.

After centrifugation, the supernatant solution was transferred to a different tube and further used for analysing the time-resolved anisotropy and CD spectra. As shown in Fig. S8a† and listed in Table S3,† the dilute phase shows a slower anisotropy decay (monitored for tryptophan emission) compared to the phase-separated solution (consisting of dilute and condensed phases) which exhibits a rapid decrease in anisotropy, and its rotational time more closely resembles that of the native protein, Fig. S6 and Table S1.† The CD spectra of the dilute phase (Fig. S8b†) indicate conformation resembling that of the phase-separated protein. Thus, we conclude that the conformational unwinding and rotational flexibility observed are the reflections of the alteration due to phase separation and condensate formation. Previously, phase separation of globular proteins has been reported to involve conformational unfolding where the conformational unwinding plays a critical role in inducing the phase separation of the native compact protein and in certain instances, partial unfolding has been suggested as a possibility.^43,44^

Further, to understand whether the unfolded conformation of the protein plays an important role in inducing such a liquid droplet state of the protein, we examined whether the phase separation would occur when the protein was denatured by guanidine hydrochloride (6 M GdHCl), a commonly used denaturant. Surprisingly, we did not observe any characteristics of liquid droplet formation by using the denatured β-LG as evident from turbidity assay (Fig. S9a†) and FESEM (Fig. S9b†). This observation highlights that the observed conformational changes are unique to the LLPS process of β-LG. It is reported that β-LG exists in dimeric form around physiological pH (∼7.4) but in the very low pH regime (pH ≤ 3), it mostly remains in its monomeric form.^45^ Therefore, we also analyzed the LLPS process of β-LG at pH ∼2.3 using FESEM images, where we did not observe any formation of condensates (Fig. S9c†), as also evident from the pH-dependent turbidity assay (Fig. S1a†). It can be rationalized based on the previous reports that at very low pH, the protein mainly exists in its monomeric form due to the excess repulsion among the individual protein chains.^45,46^ This, in turn, further results in an unfavourable condition for the condensation of multiple protein chains for the phase separation process to be effective. The lack of liquid droplets under the denatured and very low pH conditions of β-LG suggests that the phase separation and related changes are favourable near physiological pH and under phase-separation conditions using native protein.

### 3.3 Phase separation and intermolecular charge transfer state

To explore the phase separation process using the intrinsic fluorophore tryptophan, we monitored the time-dependent fluorescence emission spectra of tryptophan (*λ*_ex/em_ = 295/340 nm) in the presence of the crowder (PEG8000) incubated at 37 °C. Interestingly, during the formation of the liquid droplets, we observed an additional long-wavelength prominent emission shoulder, appearing around 400 nm (Fig. 3c). The excitation wavelength for this distinct emissive species was determined to be ∼320 nm (Fig. 3d), indicating the formation of a new emissive species. This rather unusual spectral signature has been previously reported to be originating due to an extensive intermolecular charge transfer (CT) through the hydrogen-bonded network of the polypeptide backbone enabled by trapped water molecules which is also evident from our fluorescence data in Fig. S10a.† This newly generated fluorescence peak was absent in the dispersed phase of the proteins (*i.e.*, in the absence of the LLPS process, Fig. S10b†) and gets exclusively generated as a function of time during the phase transition. It has been previously hypothesized that phase separation can be modulated by CT in the protein backbone, with an operational fluorescence resonance energy transfer (FRET) process between the intrinsic tryptophan fluorescence emission and the CT band excitation.^47^ In a similar scenario, we observed that the phase separation of β-LG is correlated with the rise of the CT band. This is supported by the data from control experiments (Fig. S10c–f†) where the CT fluorescence band was absent when LLPS was not operative. For example, when the denatured β-LG (by 6 M GdHCl) was incubated with PEG8000 and in the case of fibrillation of β-LG, no CT bands were observed (Fig. S10f†). This unique characteristic property confirms the distinctive nature of this process.

### 3.4 Effect of electrolyte and chaotropic agent on LLPS

**Fig. 4:**
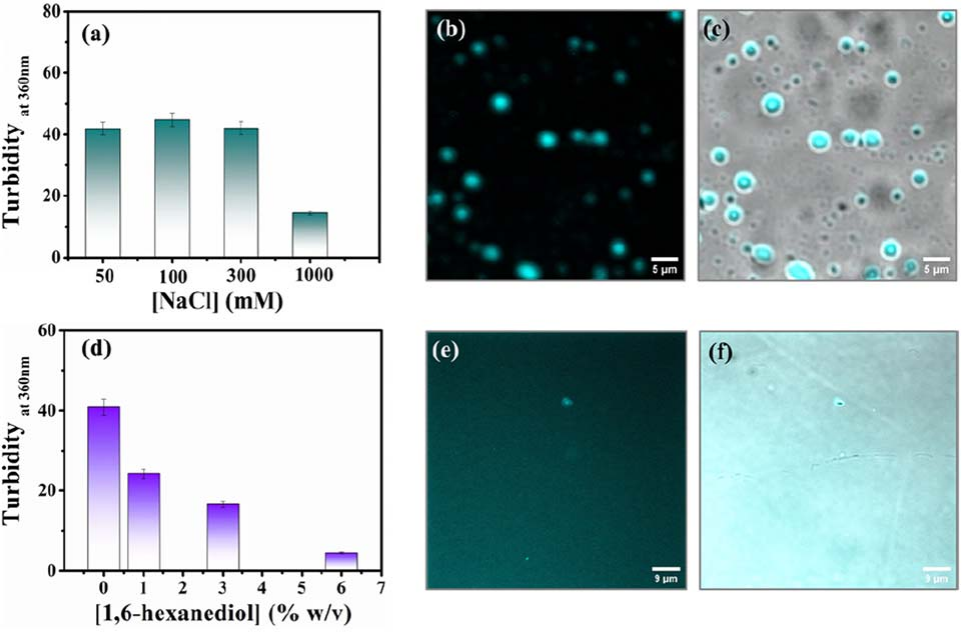
Investigating the effect of electrolyte and chaotropic agent on the LLPS of β-LG. (a) Turbidity plot of the β-LG (50 μM) incubated with a fixed concentration of PEG8000 (10% w/v) in the presence of different concentrations of NaCl. (b) Fluorescence image of the sample incubated with 300 mM of NaCl and (c) merged image combining fluorescence and phase-contrast images of the sample incubated with 300 mM of NaCl (scale bar: 5 μm). (d) Turbidity plot of the sample (50 μM β-LG in the presence of 10% w/v PEG8000, at 37 °C and pH 7.4) incubated with varying percentages of 1,6-hexanediol in the solution. (e) Fluorescence image of the sample incubated with 6% of 1,6-hexanediol in the solution and (f) merged image combining fluorescence and phase-contrast images of the sample incubated with 6% of 1,6-hexanediol in the solution (scale bar: 9 μm).

Protein-driven phase separation mainly originates from multivalent interactions between biomolecules, such as electrostatic, hydrophobic, π–π, hydrogen bonding interactions, and even through the formation of oligomers.^48^ An inert crowder like PEG8000 can prompt short-range depletion force and reduce the long-range electrostatic repulsion between biomolecules.^49,50^ To decipher the interaction mechanism behind the phase-separated liquid-like droplets of β-LG, we incubated the protein along with 10% w/v PEG8000 in solutions having different ionic strengths (50, 100, 300, and 1000 mM NaCl) and different percentages of a chaotropic agent, 1,6-hexanediol (1, 3 and 6% w/v).^1^ As evident from the turbidity data and confocal microscopic fluorescence images in Fig. 4a and b, c, respectively, the increasing concentration of NaCl (up to 300 mM) did not hinder the droplet formation process, indicating that electrostatic shielding had minimal impact on LLPS of β-LG under crowding conditions. However, at very high concentrations (∼1 M) these droplet solutions show feeble turbidity, Fig. 4a, in accordance with the previous reports.^16^

On the other hand, in the presence of an increasing concentration of 1,6-hexanediol, we observed a drastic decrease in the turbidity value (Fig. 4d) and in the presence of 6% of 1,6-hexanediol, the formation of the liquid droplets of β-LG is completely inhibited, as depicted by the fluorescence microscopy images (Fig. 4e and f). This is also reflected in FESEM (Fig. S11†) images after the serial addition of these reagents. These observations outline the predominant role of hydrophobic interactions which consequently leads to the phase separation of β-LG.

### 3.5 Polyethylene glycol as a crowder

The formation of coacervates is a synergistic effect of enthalpy and entropy of mixing.^40,51,52^ The enthalpic effect originates from the multitudes of weak non-covalent interactions between biomolecules, whereas such association is entropically favored in a crowded milieu.^53^ The presence of inert crowding agents like PEG8000 can also modulate the associated thermodynamics of biomolecules through the excluded volume effect.^54^ The excluded volume effect could reduce the conformational space for larger molecules and trigger protein–protein interactions to form liquid droplets. While proteins like lysozyme and γ-crystallins at high concentrations undergoing LLPS without the need for a crowder have been reported,^44,55^ our focus was on investigating the LLPS behaviour of β-LG at significantly lower concentrations in the presence of PEG8000. To understand the role of PEG8000, we investigated its interaction with the protein and its consequences on the photophysical responses and the secondary structure of β-LG. For this purpose, we titrated a fixed amount of the protein (50 μM for fluorescence study and 5 μM for CD) with different concentrations of the crowder PEG8000 and monitored the emission response of the intrinsic tryptophan fluorescence, which is considered to be sensitive to the perturbation of the microenvironment of the protein. As shown in Fig. S12a,† the tryptophan emission of the β-LG remains unchanged in the presence of different concentrations of PEG8000. Again, the CD spectra (Fig. S12b†) show that the presence of PEG8000, as such, has no significant/direct impact on the secondary structure of the protein. Further, to understand the possible specificity of PEG8000 for inducing LLPS, we employed dextran instead of PEG8000 at the same concentration (10% w/v) and observed the formation of droplets through DIC images of the solution (Fig. S12c†). We also observed a similar trend of change in the secondary structure with the phase separation of PEG8000 as evidenced by Fig. S12d.† To further confirm whether the droplets were composed predominantly of β-LG, or crowder (PEG) was also included inside the droplet phase, we used 5-DTAF-labelled PEG8000 for confocal fluorescence imaging. As depicted in Fig. S13a,† the droplets exhibit no prominent fluorescence intensity of the PEG8000 labelled with 5-DTAF compared to the bulk solution in contrast to the imaging with CPM-labelled β-LG which shows distinct fluorescent droplets (Fig. 2b–d, 4b and S13b, c†) re-emphasizing the notion of protein-rich condensates. The line intensity plot (Fig. S13d†) further substantiates the negligible intensity of PEG8000-labelled 5-DTAF within the droplets. These observations collectively indicate the role of PEG8000 as an inert crowder with no specific interaction with β-LG and exerts an excluded volume effect for inducing phase separation of β-LG. Theoretically, it has been proposed that π–π interactions between amino acids drive the LLPS phenomenon^51,56^ and it is possible that the crowded environment within the condensed phase could facilitate unfolding of individual proteins.^44^

### 3.6 Effect of LLPS on esterase-like activity of the protein

**Fig. 5:**
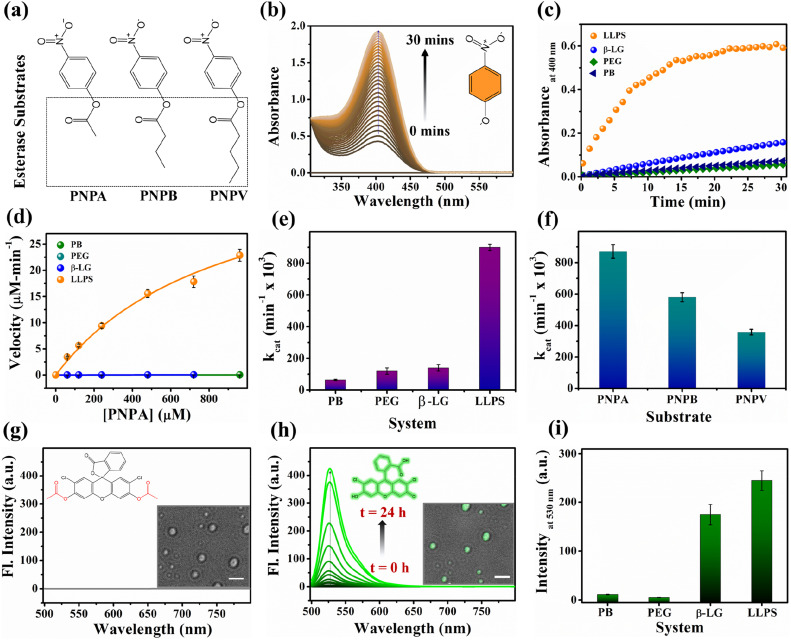
The functional activity of the protein under native and phase-separated conditions. (a) Structural representation of the ester substrates PNPA, PNPB, and PNPV used for analysis. (b) Gradual enhancement in the absorbance peak around 400 nm for the hydrolyzed product, PNP in the presence of liquid–liquid phase-separated β-LG. (c) Esterase kinetics of PNPA hydrolysis (keeping [PNPA] = 120 μM; monitoring absorbance at 400 nm) by β-LG condensates (50 μM β-LG in 10% w/v PEG8000, at 37 °C and pH 7.4 after 7 days) compared with the native protein (50 μM), PEG8000 (10% w/v), and buffer as controls. (d) Comparative analysis of the rate of hydrolysis of PNPA as a function of its concentration by β-LG condensates, the native protein, PEG8000, and phosphate buffer. (e) Comparative analysis of the catalytic rate constant (*k*_cat_) for PNPA hydrolysis using β-LG condensates, the native protein, PEG8000, and phosphate buffer under similar conditions (having PNPA substrate concentration of 120 μM, 50 μM β-LG, 10% w/v PEG8000). (f) Comparative analysis of the catalytic rate constant (*k*_cat_) for different substrates, *viz.* 120 μM PNPA, PNPB, and PNPV under similar conditions (50 μM β-LG, 10% w/v PEG8000; temperature 37 °C and pH 7.4) using liquid–liquid phase-separated β-LG. (g) Fluorescence emission spectra of DCFDA when excited at 490 nm; the inset structure represents the chemical structure of the non-emissive DCFDA and the image represents the confocal micrograph of the droplets immediately after the addition of a fresh solution of DCFDA (scale bar: 3 μm). (h) Fluorescence emission spectra of DCF (*λ*_ex/em_ = 490/530 nm) monitored as a function of time; the inset structure represents the chemical structure of the hydrolyzed product DCF which is emissive and the image represents the confocal micrograph of the droplets (brightfield merged with the fluorescence image) after 24 h hydrolysis of DCFDA (scale bar: 3 μm). (i) Comparative analysis of the fluorescence emission of DCF formed through the different extent of hydrolysis from β-LG condensates, native protein, PEG8000, and phosphate buffer under similar conditions.

Proteins are known for their important functions in physiology, including their smart enzymatic activities. Thus, it becomes vital to understand the alterations in the functional properties of the protein in various stages of LLPS. After thorough characterization by spectroscopic, morphological, and conformational properties, we strived to look for the alteration in functional properties of the β-LG inside such liquid-droplet. β-LG, a whey protein, is not popularly known to exhibit esterase-like activity and its biological functions are not entirely clear, although there are some suggestions.^57^ Hence, in order to understand the modulation in the functional properties, we investigated the esterase activity profile before and after the phase separation of β-LG. For this purpose, we primarily selected p-nitro phenyl acetate (PNPA), a standard substrate (shown in Fig. 5a). The esterase enzymatic activity of the protein in the phase-separated state was monitored by measuring the changes in absorbance values at 400 nm (Fig. 5b) corresponding to the hydrolyzed product, *p*-nitrophenolate (PNP). As can be seen from Fig. 5c and d, the autohydrolysis of PNPA seems to be extremely slow and the presence of PEG8000 also doesn't alter our observations. Surprisingly, while β-LG showed almost no catalytic activity towards ester hydrolysis in its native state, the phase-separated liquid-like condensate exhibited efficiently high kinetics, suggesting the emergence of a promiscuous esterase-like activity of β-LG post phase separation. The formation of PNP as the hydrolyzed product was confirmed using the ESI-MS technique as depicted by the *m*/*z* peak centered at 138.0391 in the mass spectra (Fig. S14†). The esterase activity of the phase-separated proteins is easily distinguished from the hydrolysis of PNPA in phosphate buffer, PEG8000, and native β-LG solution (Fig. S15a–c†). The catalytic rate constant (*k*_cat_) and Michaelis constant (*K*_m_) were estimated from multiple measurements by varying concentrations of the substrates (Fig. S15a–d†) and from the Michaelis–Menten enzyme kinetic analysis using nonlinear curve fitting (Fig. 5d, Table S4†). This shows that the characteristic of the enzymatic activity towards PNPA is exclusively due to the phase-separated β-LG (Fig. 5e). To further explore the substrate scope and generalization of the observed enzymatic activity, we verified the esterase activity of the phase-separated system with two more chromogenic esterase substrates (Fig. S15e and f†): *p*-nitrophenyl butyrate (PNPB) and *p*-nitrophenyl valerate (PNPV). The kinetics analyses in the presence of phase-separated β-LG displayed similar catalytic behaviour for these substrates as well, as observed for PNPA (Fig. S16a and b†). As expected, native β-LG was non-catalytic towards the hydrolysis of PNPB and PNPV, and PEG8000 alone has no role in the enzymatic activity, exhibiting a similar absorbance as encountered in the autohydrolysis process (*i.e.*, in PB/water alone). The catalytic rate constant (*k*_cat_) and Michaelis constant (*K*_m_) for the PNPB and PNPV hydrolysis reaction were obtained similarly from the Michaelis–Menten enzyme kinetic analysis (Fig. S17a and b†) and are represented by Fig. 5f and listed in Table 1.

**Table 1:** The functional activity of the phase-separated protein for various ester substrates

Substrate	*V*_max_ (μM min^−1^)	*K*_m_ (μM)	*k*_cat_ (min^−1^ × 10^3^)
PNPA	43 ± 2	900 ± 50	880 ± 40
PNPB	29 ± 2	240 ± 10	570 ± 30
PNPV	18 ± 1	250 ± 10	360 ± 20

Furthermore, we conducted a qualitative analysis of the enzymatic capability of β-LG in the phase-separated state using another esterase substrate, DCFDA, by fluorescence spectroscopy. As can be seen from Fig. 5g–i, the systematic enhancement in the fluorescence intensity with time confirmed that the phase-separated liquid droplets of β-LG can also catalyze the hydrolysis of non-fluorescent DCFDA to release the fluorescent product DCF (Fig. 5h).

We conducted similar studies on two different globular proteins, HSA and BSA, under identical conditions. The DIC images (Fig. S18a and b†) revealed the formation of condensates. However, the analysis of the secondary structure through CD spectra (Fig. S18c and d†) showed no significant alteration, consistent with previous findings.^21,22^ Subsequently, we performed a PNPA hydrolysis experiment in the presence of native and phase-separated HSA and BSA. As evident from Fig. S18e and f,† no notable modulation in esterase-like activity was observed for either HSA or BSA. This indicates that the modulation in one kind of functional property may not be universal to all the proteins as the alteration in the esterase activity can be seen for phase-separated β-LG, but not for other globular proteins tested here (*i.e.*, HSA and BSA). Further, we investigated whether amyloidogenic aggregation or denaturation of β-LG can regulate such modulations in enzymatic activity. For this purpose, we conducted a PNPA kinetics experiment with aggregated β-LG as well as with a denatured sample of β-LG (by 6 M GdHCl) incubated in the presence of PEG8000 (Fig. S19†). We also conducted sequential denaturation of the protein (partial to complete denaturation) using various denaturing agents (*viz.* 0–6 M GdHCl, and 0–8 M urea) followed by monitoring the secondary structure through CD and conducting PNPA kinetics (Fig. S20†). Notably, the activity observed in phase-separated β-LG was absent in the amyloid and denatured forms of β-LG (Fig. S19, S20b, and S20d†) emphasizing the distinct influence of phase separation on enzymatic activity.

To gain additional insights into the enzymatic activity within the phase-separated protein, we conducted separate PNPA hydrolysis experiments in the dilute phase. Inspired by a previous report we conducted centrifugation followed by separation of the dilute phase for further study.^58^ As shown in Fig. S21,† the dilute phase displayed negligible activity, almost similar to the native protein. These findings strongly support the idea that the condensates are responsible for the catalytic activity. Additionally, we found that these droplet-like condensates remain intact even after the enzymatic activity, as observed in the DIC images in Fig. S22a† and the inset image of Fig. 5g and h. Therefore, taking advantage of this, we acquired the confocal microscopy images of the phase-separated solution after the hydrolysis of DCFDA. Interestingly, the bright field images of the droplets exhibited a prominent and distinct bright green fluorescence (Fig. S22a–c† and the inset image of Fig. 5h). This compelling evidence directly indicates the presence of a catalytic site for ester hydrolysis within these highly dense protein droplets, further reinforcing the concept that the condensates act as robust catalytic crucibles for the esterase hydrolysis reaction.

These comprehensive observations firmly establish that the phase-separation of the protein significantly modulates (gives rise to promiscuous function) the enzymatic activity compared to the native state of the protein. The condensates formed by phase separation create an ideal environment for efficient ester hydrolysis, resulting in the enhanced cleavage of esters.

This enzymatic activity within the liquid-like droplets highlights the unique functional properties that can emerge in such membrane-less compartments, providing valuable insights into the functional roles of protein phase separation in cellular processes. Here, the excluded volume effect in the presence of PEG8000 can modulate the effective concentrations of β-LG compared to the global concentrations that could play a seminal role in the enzymatic activity shown by the β-LG rich liquid droplets herein.^59^ The nonstoichiometric bio-assemblies have been reported to execute good enzymatic activity.^60^ In addition, the different chemical environments and conformational organization of proteins inside the liquid-like droplets could create a conducive chemical environment by exposing amino acids to catalyze the hydrolysis reaction. In esterase-like enzymatic reactions, specific amino acids such as tyrosine (Tyr), arginine (Arg), serine (Ser), histidine (His), and glutamic acid (Glu)/aspartic acid (Asp) in proteins and peptides have been known to participate in catalytic activities by interacting with substrates.^61,62^ Hence, the presence of these amino acids in β-LG supports its potential for demonstrating esterase-like enzymatic activity. While serine proteases are well-known to form catalytic triads involving Ser, Asp/Glu, and His residues,^61,63^ there are reports of esterase activity operating through different mechanisms as well.^64,65^

The involvement of water molecules and proton transfer in enzymatic reactions is a critical aspect for understanding the catalytic mechanisms (since, its result is the reflection of the acetylation reaction that occurs during the first step of the hydrolytic reaction, generating *p*-nitrophenol).^61,66^ Serine proteases, for instance, exhibit a solvent isotope effect dependent on the mole fraction of deuterium oxide, indicating the direct involvement of the active-site serine hydroxyl group and water molecules in their catalytic triads.^61^ Hence, we attempted to evaluate the role of water molecules and proton transfer using the solvent isotope effect. To this end, we performed PNPA hydrolysis kinetics in solutions having 0% and 50% D_2_O as solvent. Our experimental result demonstrated that the presence of deuterium oxide has no significant effect on the catalytic profile of the enzyme (phase separated β-LG here, Fig. S23†). This finding aligns with a previous report (of hydrolysis catalyzed by the protein HSA)^62^ where the lack of isotope effect indicates that proton transfer is not a major factor during the initial step of the hydrolytic reaction. This observation implies that the probable catalytic mechanism of our protein is not dependent on general acid or base catalysis, as is the case for serine proteases. Instead, it suggests that the catalytic process is primarily driven by other factors, such as specific amino acid residues within the active site as described for albumins. The significance of tyrosine and arginine in hydrolytic activity is evident in the case of albumins (HSA),^61^ as they are perfectly preserved in all species of albumin, and the differences in reactivity among different albumins are believed to reflect differences in the microenvironment.^61,66–68^ This suggests that the catalytic process is most likely driven by specific amino acid residues within or in the vicinity of the active site. We also analyzed the PDB structure of the β-LG (PDB ID:1BEB) using PyMOL, for the availability of these residues on the protein surface. We found that β-LG has 4 tyrosine (3 present in β-strands: Y20, Y42, Y102) and 3 arginine (1 present in the β-strand: R148) (Fig. S24†). To substantiate our findings, we conducted PNPA kinetics experiment in the presence and absence of a medium-chain fatty acid, caproic acid (CA). Intriguingly, we observed that even at a low concentration (0.0003% w/v), CA profoundly inhibits the esterase-like activity (Fig. S25†). This inhibition pattern further supports the notion of a mechanism, analogous to the albumin esterases.^66–68^ Proteins possess diverse conformations and activities beyond their primary roles.^69,70^ Our systematic day-wise study indicates that the catalytic activity increases (Fig. S26†) as the β-sheet content decreases with phase separation (Fig. 3b). 2,2,2-Trifluoroethanol (TFE) can prompt a shift in the conformational distribution of β-LG, transitioning it from being predominantly composed of β-sheets to having a higher proportion of α-helices.^71^ This led us to deliberately modify the conformational distribution of β-LG using TFE, which we monitored through detailed CD measurements (shown in Fig. S27a–c,† and detailed in the corresponding figure caption). Further, we delved into examining how this altered conformational state of β-LG affected its esterase-like activity. Interestingly, we found that the shift in conformational distribution is accompanied by changes in the esterase-like enzymatic activity of the protein. This strongly suggests a correlation between conformational changes and functional diversity (Fig. S28a and b†).

The observed modulation in esterase-like activity under the influence of TFE suggests that conformational alterations (and stabilization of the α-helix-rich conformer), rather than complete denaturation, trigger this promiscuous function. This resonates with the concept of promiscuous functions emerging from alternate conformations.^69,71^ This thereby elucidates the intricate relationship between protein conformational states and functional activities and highlights how the shift in the conformational distributions under specific conditions, such as phase separation, can markedly influence protein functionality.

This finding adds to the growing body of research that certain proteins may employ distinct catalytic strategies, contributing to the functional diversity observed in enzymatic reactions. Further investigations into the specific residues and structural motifs responsible for this unique enzymatic activity will shed more light on the fascinating world of protein catalysis. Our present investigation thus substantiates that chemical environments and conformational changes play a pivotal role in concentrating the substrate within condensates to catalyse the hydrolysis reaction.

## 4. Conclusions

Our study provides comprehensive insights into the phase separation behavior of β-LG and the formation of liquid-like droplets induced by an inert synthetic crowder, PEG8000. Through a series of spectroscopic and microscopic experiments, we have demonstrated the formation of liquid-like droplets, distinct from fibrillation or amyloidogenic aggregation, predominantly driven through hydrophobic interactions. These droplets exhibit dynamic behavior consistent with liquid-like properties, characterized by confocal fluorescence microscopy and FRAP study. Time-resolved anisotropy and CD investigations revealed how LLPS modulates the conformational flexibility of β-LG, subsequently inducing conformational changes. The presence of a charge transfer (CT) state, in the case of phase-separated protein, further confirmed the unique nature of this process. Interestingly, phase separation alters the functional activity of β-LG, giving rise to esterase enzymatic activity compared to the native state as verified with various chromogenic and fluorogenic esterase substrates. Notably, such modulation was specific to the phase-separated state, a unique characteristic not observed in the denatured or aggregated state of β-LG and also not seen in other globular proteins (BSA and HSA). We found that the droplets harbour a catalytic site and act as robust crucibles for esterase hydrolysis, likely driven by specific amino acid residues exposed through phase separation, similar to albumins. The promiscuous function emerging from alternate conformations elucidates how the shift in the conformational distributions under specific conditions, such as phase separation, can unveil the latent functionality. Further investigation into these residues and structural motifs may shed more light on the protein catalysis mechanism. These findings highlight the functional properties that can emerge in membrane-less compartments due to phase separation, providing valuable insights into the roles of protein phase separation in cellular processes.

## Data availability

All the experimental and characterization data are available in the manuscript and ESI.†

## Author contributions

SR and SM conceptualized the project, SR and SP carried out the experiments, SR mainly analyzed the data, all authors were involved in writing the manuscript.

## Conflicts of interest

There are no conflicts to declare.

## Supplementary Material

SC-015-D3SC06802A-s001

SC-015-D3SC06802A-s002

## References

[cit1] Mimura M., Tomita S., Shinkai Y., Hosokai T., Kumeta H., Saio T., Shiraki K., Kurita R. (2021). J. Am. Chem. Soc..

[cit2] Fisher R. S., Elbaum-Garfinkle S. (2020). Nat. Commun..

[cit3] Zhang Y., Yang M., Duncan S., Yang X., Abdelhamid M. A. S., Huang L., Zhang H., Benfey P. N., Waller Z. A. E., Ding Y. (2019). Nucleic Acids Res..

[cit4] Patel A., Lee H. O., Jawerth L., Maharana S., Jahnel M., Hein M. Y., Stoynov S., Mahamid J., Saha S., Franzmann T. M. (2015). et al.. Cell.

[cit5] Banani S. F., Lee H. O., Hyman A. A., Rosen M. K. (2017). Nat. Rev. Mol. Cell Biol..

[cit6] Dignon G. L., Best R. B., Mittal J. (2020). Annu. Rev. Phys. Chem..

[cit7] Vernon R. M., Chong P. A., Tsang B., Kim T. H., Bah A., Farber P., Lin H., Forman-Kay J. D. (2018). eLife.

[cit8] Das S., Lin Y. H., Vernon R. M., Forman-Kay J. D., Chan H. S. (2020). Proc. Natl. Acad. Sci. U. S. A..

[cit9] Portz B., Lee B. L., Shorter J. (2021). Trends Biochem. Sci..

[cit10] Wang B., Zhang L., Dai T., Qin Z., Lu H., Zhang L., Zhou F. (2021). Signal Transduction Targeted Ther..

[cit11] Shin Y., Brangwynne C. P. (2017). Science.

[cit12] Boeynaems S., Alberti S., Fawzi N. L., Mittag T., Polymenidou M., Rousseau F., Schymkowitz J., Shorter J., Wolozin B., Van Den Bosch L., Tompa P., Fuxreiter M. (2018). Trends Cell Biol..

[cit13] Reichheld S. E., Muiznieks L. D., Keeley F. W., Sharpe S. (2017). Proc. Natl. Acad. Sci. U. S. A..

[cit14] Malay A. D., Suzuki T., Katashima T., Kono N., Arakawa K., Numata K. (2020). Sci. Adv..

[cit15] Maier R., Zocher G., Sauter A., Da Vela S., Matsarskaia O., Schweins R., Sztucki M., Zhang F., Stehle T., Schreiber F. (2020). Cryst. Growth Des..

[cit16] Wegmann S., Eftekharzadeh B., Tepper K., Zoltowska K. M., Bennett R. E., Dujardin S., Laskowski P. R., Mackenzie D., Kamath T., Commins C. (2018). et al.. Eng. Med..

[cit17] Ray S., Singh N., Kumar R., Patel K., Pandey S., Datta D., Mahato J., Panigrahi R., Navalkar A., Mehra S. (2020). et al.. Nat. Chem..

[cit18] Ambadipudi S., Biernat J., Riedel D., Mandelkow E., Zweckstetter M. (2017). Nat. Commun..

[cit19] Darling A. L., Liu Y., Oldfield C. J., Uversky V. N. (2018). Proteomics.

[cit20] Uversky V. N., Kuznetsova I. M., Turoverov K. K., Zaslavsky B. (2015). FEBS Lett..

[cit21] Poudyal M. , Patel K., Sawner A. S., Gadhe L., Kadu P., Datta D., Mukherjee S., Ray S., Navalkar A., Maiti S., et al., *bioRxiv*, 2021, preprint, 10.1101/2021.12.31.474648

[cit22] Patel C. K., Singh S., Saini B., Mukherjee T. K. (2022). J. Phys. Chem. Lett..

[cit23] Ghosh N., Mondal R., Mukherjee S. (2015). Langmuir.

[cit24] Liu L., Michelsen K., Kitova E. N., Schnier P. D., Klassen J. S. (2012). J. Am. Chem. Soc..

[cit25] Paul B. K., Ghosh N., Mukherjee S. (2014). Langmuir.

[cit26] Kontopidis G., Holt C., Sawyer L. (2004). J. Dairy Sci..

[cit27] Rodrigues L. R., Venâncio A., Teixeira J. A. (2001). Biotechnol. Lett..

[cit28] Trajkovski M., Endoh T., Tateishi-Karimata H., Ohyama T., Tanaka S., Plavec J., Sugimoto N. (2018). Nucleic Acids Res..

[cit29] Biswas S., Mukherjee S. K., Chowdhury P. K. (2016). J. Phys. Chem. B.

[cit30] Das N., Sen P. (2018). Biochemistry.

[cit31] Pramanik S., Chithra S., Rai S., Agrawal S., Shil D., Mukherjee S. (2023). J. Phys. Chem. B.

[cit32] Pramanik U., Khamari L., Rai S., Mahato P., Nandy A., Yadav R., Agrawal S., Mukherjee S. (2022). ChemPhysChem.

[cit33] Pramanik S., Khamari L., Mukherjee S. (2021). J. Phys. Chem. Lett..

[cit34] Apte A., Manich M., Labruyère E., Datta S. (2022). PLoS Pathog..

[cit35] Khamari L., Pramanik U., Shekhar S., Mohanakumar S., Mukherjee S. (2021). Langmuir.

[cit36] Roquero D. M., Bollella P., Katz E., Melman A. (2021). ACS Appl. Polym. Mater..

[cit37] Khin M. N., Ahammed S., Zhong F. (2021). Food Biosci..

[cit38] Jones O. G., Adamcik J., Handschin S., Bolisetty S., Mezzenga R. (2010). Langmuir.

[cit39] Taylor N. O., Wei M. T., Stone H. A., Brangwynne C. P. (2019). Biophys. J..

[cit40] Majumdar A., Dogra P., Maity S., Mukhopadhyay S. (2019). J. Phys. Chem. Lett..

[cit41] Greenfield N. J. (2006). Nat. Protoc..

[cit42] Le Maux S., Bouhallab S., Giblin L., Brodkorb A., Croguennec T. (2014). Dairy Sci. Technol..

[cit43] Ruff K. M., Choi Y. H., Cox D., Ormsby A. R., Myung Y., Ascher D. B., Radford S. E., Pappu R. V., Hatters D. M. (2022). Mol. Cell.

[cit44] Cinar S., Cinar H., Chan H. S., Winter R. (2019). J. Am. Chem. Soc..

[cit45] Khan S., Ipsen R., Almdal K., Svensson B., Harris P. (2018). Biomacromolecules.

[cit46] Sakurai K., Oobatake M., Goto Y. (2001). Protein Sci..

[cit47] Dogra P., Joshi A., Majumdar A., Mukhopadhyay S. (2019). J. Am. Chem. Soc..

[cit48] Tourriere H., Chebli K., Zekri L., Courselaud B., Blanchard J. M., Bertrand E., Tazi J. (2003). J. Cell Biol..

[cit49] Marenduzzo D., Finan K., Cook P. R. (2006). J. Cell Biol..

[cit50] Groen J., Foschepoth D., te Brinke E., Boersma A. J., Imamura H., Rivas G., Heus H. A., Huck W. T. (2015). J. Am. Chem. Soc..

[cit51] Posey A. E., Holehouse A. S., Pappu R. V. (2018). Methods Enzymol..

[cit52] Martin E. W., Mittag T. (2018). Biochemistry.

[cit53] André A. A. M., Spruijt E. (2020). Int. J. Mol. Sci..

[cit54] Kaur T., Alshareedah I., Wang W., Ngo J., Moosa M. M., Banerjee P. R. (2019). Biomolecules.

[cit55] Cinar H., Fetahaj Z., Cinar S., Vernon R. M., Chan H. S., Winter R. H. A. (2019). Chem.–Eur. J..

[cit56] Dannenhoffer-Lafage T., Best R. B. (2021). J. Phys. Chem. B.

[cit57] Tai C. S., Chen Y. Y., Chen W. L. (2016). BioMed Res. Int..

[cit58] Peeples W., Rosen M. K. (2021). Nat. Chem. Biol..

[cit59] Minton A. P. (2001). J. Biol. Chem..

[cit60] Zhao X., Palacci H., Yadav V., Spiering M. M., Gilson M. K., Butler P. J., Hess H., Benkovic S. J., Sen A. (2018). Nat. Chem..

[cit61] Sakurai Y., Ma S. F., Watanabe H., Yamaotsu N., Hirono S., Kurono Y., Kragh-Hansen U., Otagiri M. (2004). Pharm. Res..

[cit62] Levisson M., van der Oost J., Kengen S. W. (2007). FEBS J..

[cit63] Stein R. L. (1983). J. Am. Chem. Soc..

[cit64] Ohta N., Kurono Y., Ikeda K. (1983). J. Pharm. Sci..

[cit65] Means G. E., Bender M. L. (1975). Biochemistry.

[cit66] (a) Fersht A. , Structure and Mechanism in Protein Science, Freeman, New York, 1998

[cit67] Rabbani G., Ahn S. N. (2019). Int. J. Biol. Macromol..

[cit68] Watanabe H., Tanase S., Nakajou K., Maruyama T., Kragh-Hansen U., Otagiri M. (2000). Biochem. J..

[cit69] Sikosek T., Chan H. S. (2014). J. R. Soc., Interface.

[cit70] Amitai G., Gupta R. D., Tawfik D. S. (2007). HFSP J..

[cit71] Hamada D., Segawa S., Goto Y. (1996). Nat. Struct. Biol..

