# Supplementary material for: Deciphering the liquid–liquid phase separation induced modulation in the structure, dynamics, and enzymatic activity of an ordered protein β-lactoglobulin
Source: Chem Sci. 2024 Feb 8;15(11):3936–48. doi: 10.1039/d3sc06802a (PMC10935713; doi:10.1039/d3sc06802a)
Supplement: SC-015-D3SC06802A-s001 [file SC-015-D3SC06802A-s001.pdf]

Electronic Supplementary Material (ESI) for Chemical Science.
This journal is © The Royal Society of Chemistry 2024

Supporting Information

Deciphering the liquid-liquid phase separation induced modulation in the structure, dynamics, and enzymatic activity of an ordered protein β-lactoglobulin

Saurabh Rai[a], Srikrishna Pramanik[a] and Saptarshi Mukherjee*[a]

[a] *Department of Chemistry,*
Indian Institute of Science Education and Research Bhopal, Bhopal Bypass Road,
Bhopal 462 066, Madhya Pradesh, India.
Email: saptarshi@iiserb.ac.in

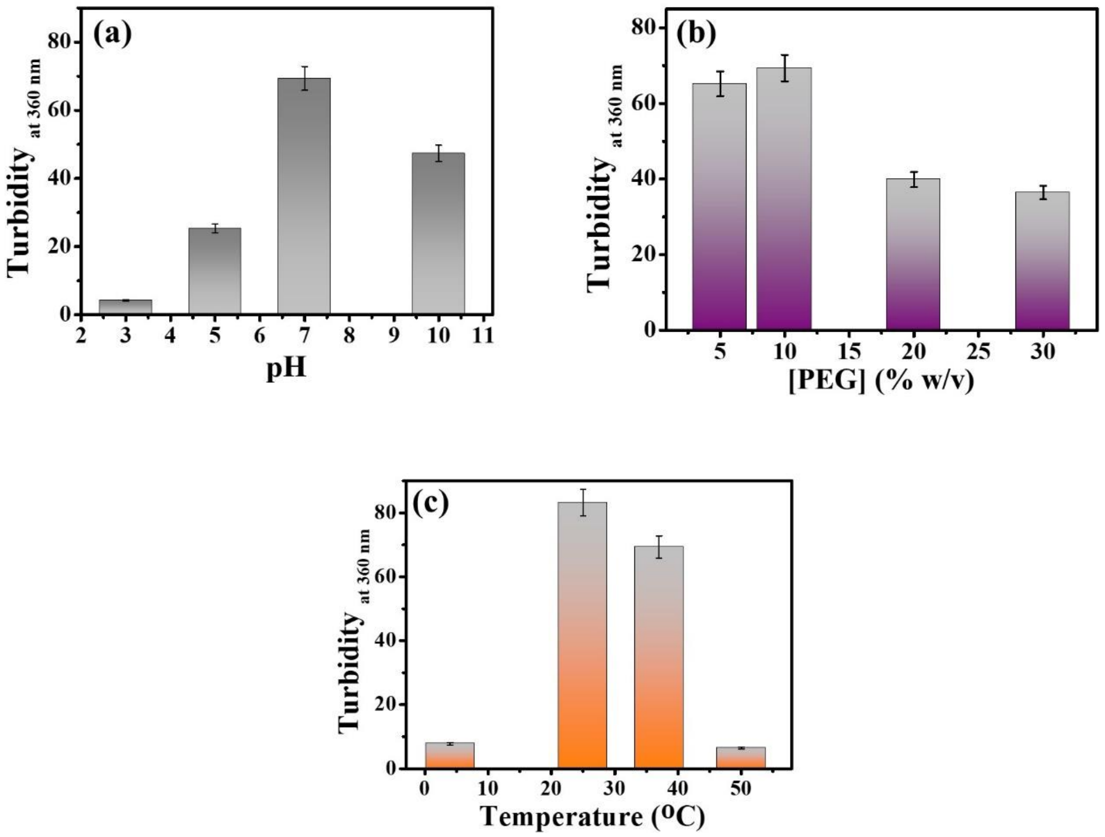

Fig. S1 Turbidity analysis for assessing the optimum phase separation conditions. (a) Turbidity of β-LG (50 µM) incubated with PEG8000 (10% w/v) under different pH of the medium, (b) under different concentrations of PEG8000 in solution keeping the concentration of β-LG fixed at 50 µM and pH 7.4, and (c) at different temperatures of incubation (using 50 µM of β-LG and 10% w/v PEG8000).

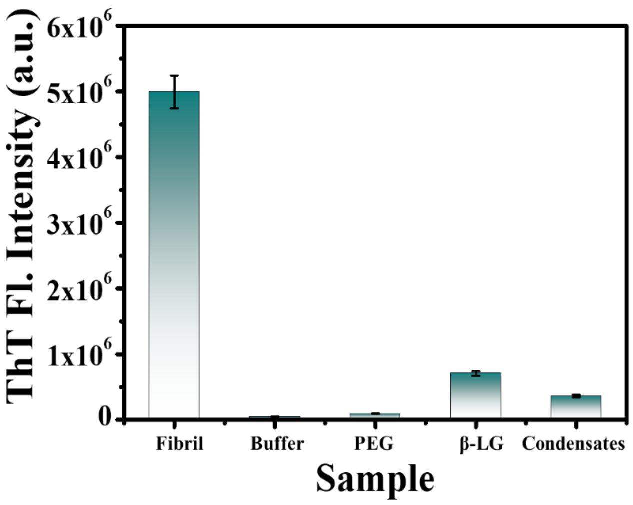

Fig. S2 Investigation of aggregation possibility in the system and comparison of droplets with fibrillar aggregates. ThT fluorescence assay of condensates (50 µM β-LG incubated with 10% w/v PEG8000 at pH 7.4 and temperature 37 °C) as compared to the aggregated protein along with the native protein, PEG8000 and PB.

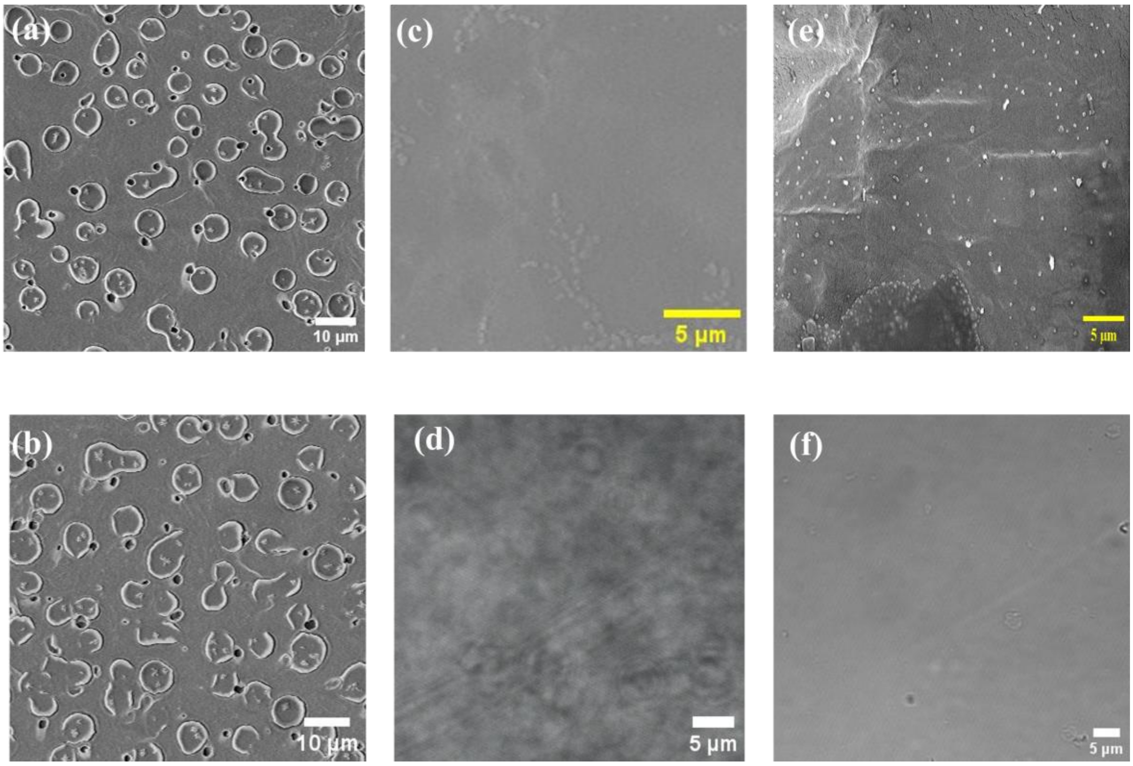

Fig. S3 Microscopic analysis for the control experiments to decipher the role of microenvironment for the LLPS process. (a) and (b) represent the FESEM images of β-LG condensates (50 µM β-LG incubated with 10% w/v PEG8000 at 37 °C and pH 7.4) showing coalescence and surface wettability, respectively. (c) and (d) represent FESEM and DIC images, respectively of only protein (50 µM β-LG) incubated at 37 °C and pH 7.4 after 7 days. (e) and (f) represent FESEM and DIC images, respectively of PEG8000 (10% w/v) incubated without protein at 37 °C and pH 7.4 after 7 days.

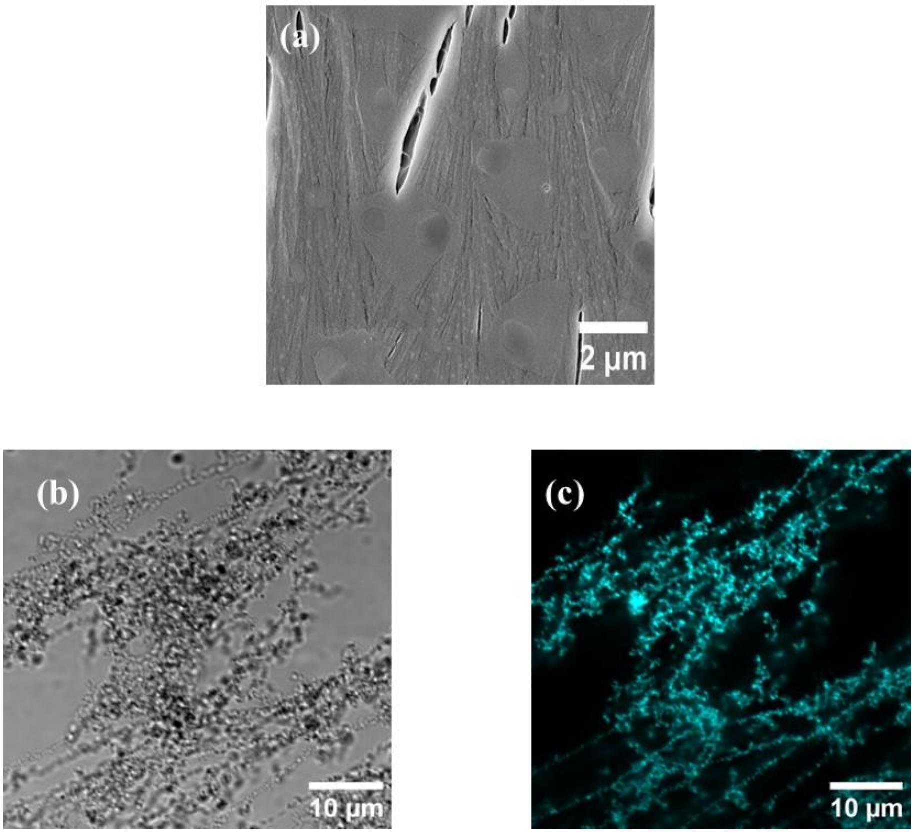

Fig. S4 Microscopic images of fibrillar aggregates of the protein, β-LG. (a) FESEM image, (b) DIC image, and (c) Confocal microscopic fluorescence image of the fibrils of β-LG, formed through incubation of β-LG at 90 °C for 6 h.

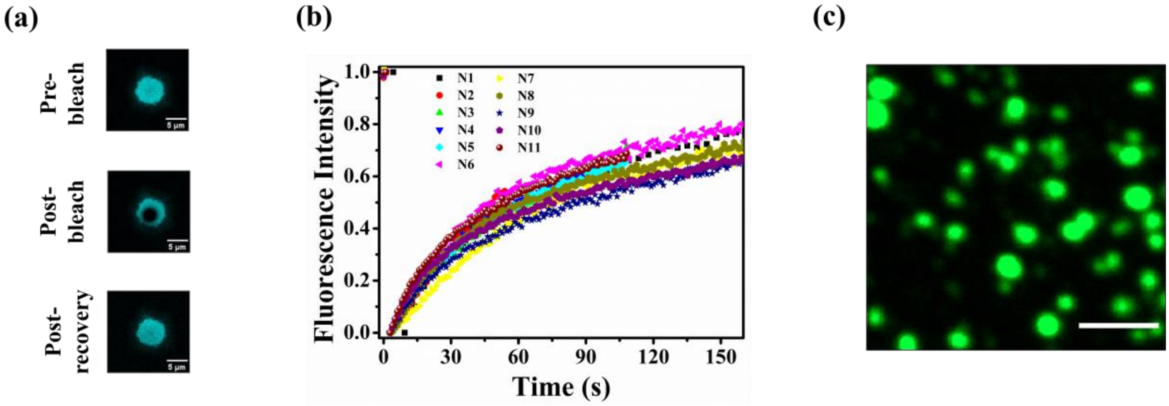

Fig. S5 (a) FRAP experiments images (pre-bleach, post-bleach, and post-recovery) performed by bleaching the droplets towards edges and (b) the corresponding FRAP kinetics recorded for 11 independent experiments. The sample used for FRAP analysis was prepared by incubating 50 μM β-LG (having 5% CPM-labelled β-LG) with PEG8000 (10% w/v) at pH 7.4 and temperature 37 °C for 5 days. (c) Fluorescence images of Rh6G-partitioned liquid droplets of β-LG (excitation laser 532 nm, scale bar: 3 μm); The concentration of Rh6G was kept at 1 μM in the condensate solution prepared by incubation of 50 μM β-LG with 10% w/v PEG8000 at pH 7.4 and temperature 37 °C for 7 days.

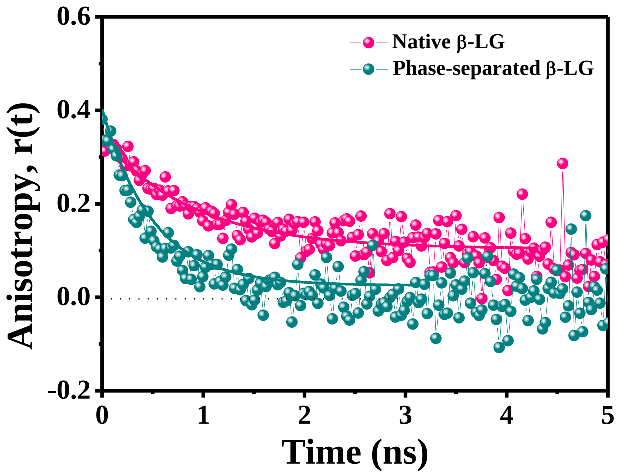

Fig. S6 The conformational analysis of the protein through time-resolved anisotropy measurements monitoring rotational dynamics of intrinsic rotor-tryptophan. The time-resolved anisotropy decay for the protein solution incubated at 37 °C and pH 7.4 in the absence and presence of PEG8000 (10% w/v) after day 7.

Table S1 Time-resolved anisotropy parameters obtained by monitoring the best chi-square fit for the anisotropy decay profiles (fitted to exponential decay functions) of LLPS system and control for tryptophan as a molecular rotor. The sample contains β-LG incubated at a temperature 37 °C and pH 7.4 in the absence and presence of PEG8000 (10% w/v) after day 7. r_0 represents the initial anisotropy.

System	r_0	$<\tau>_{rot}{}^{\Psi}$ (ns)
Phase-separated β-LG	0.38	0.5
Native β-LG	0.33	0.8

$^{\Psi}$ = ±5%

Table S2 Time-resolved anisotropy parameters obtained by monitoring the best chi-square fit for the anisotropy decay profiles (fitted to bi-exponential decay functions) of LLPS system and different controls for CPM (labeled to β-LG) as a molecular rotor. The samples contain 5% CPM-labelled β-LG along with unlabelled β-LG incubated at a temperature 37 °C and pH 7.4 in the presence of PEG8000 (10% w/v) immediately after addition, after day 7, and native protein without PEG8000.

System	r_0	α_1	τ_1 (ns)	α_2	τ_2 (ns)	$\langle\tau\rangle_{rot}^{\Psi}$ (ns)
Phase-separated β-LG (Day 7)	0.23	0.26	0.46	0.74	1.47	1.21
β-LG in PEG8000 (Day 0)	0.24	0.01	0.19	0.99	13.19	13.09
Native β-LG	0.22	0.01	0.15	0.99	12.62	12.52

$^{\Psi}$ = ±5%

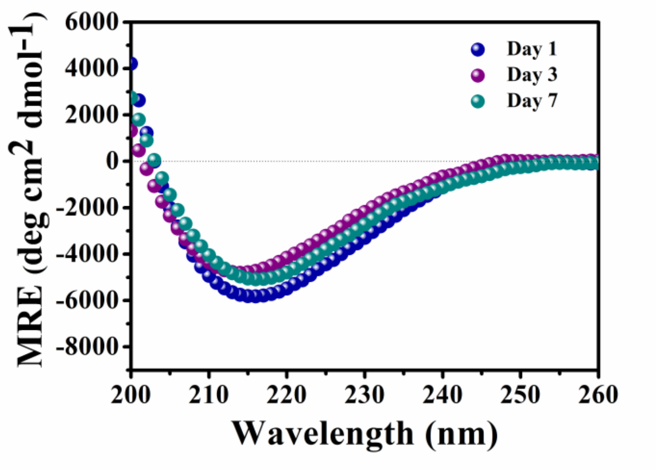

Fig. S7 Day-wise CD spectra of the protein incubated without PEG8000. Samples for recording the spectra were prepared by diluting the condensate solution (incubated 50 µM β-LG at a temperature 37 °C and pH 7.4 in PB).

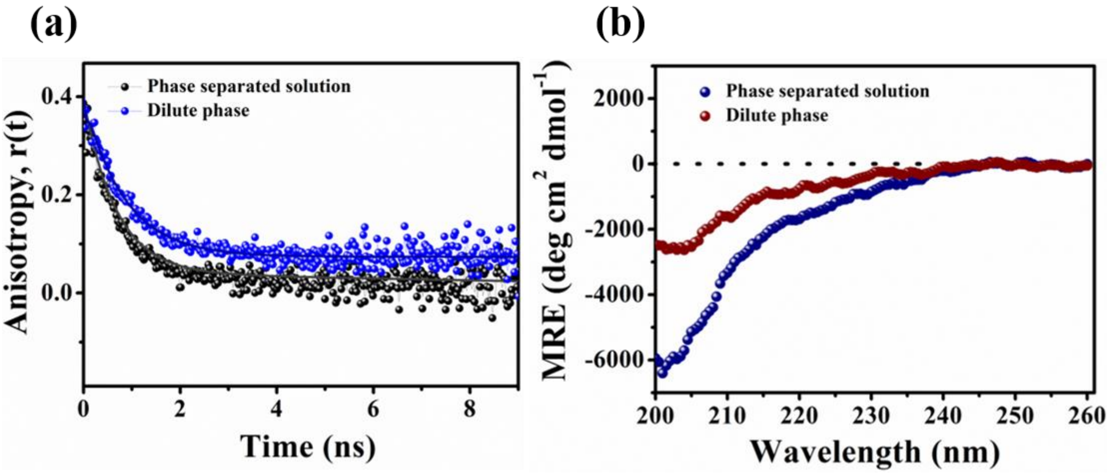

Fig. S8 Investigation of the rotational and conformational dynamics in the total phase separated solution compared to the dilute phase. (a) Anisotropy decay of the protein measured using intrinsic fluorophore, after centrifugation and separation of the dilute phases from condensates. The samples for centrifugation consisted of 50 µM β-LG incubated in the presence of PEG8000 (10% w/v) at a temperature 37 °C and pH 7.4 for 7 days. (b) CD spectra of the protein, after centrifugation and separation of the dilute phases. (Sample: 50 µM β-LG incubated with 10% w/v PEG8000, at 37 °C and pH 7.4 in 10 mM PB).

Table S3 Time-resolved anisotropy parameters obtained by monitoring the best chi-square fit for the anisotropy decay profiles (fitted to exponential decay functions) after centrifugation and separation of dilute phase and dense phase. The samples for centrifugation consisted of 50 µM β-LG incubated with PEG8000 (10% w/v) at a temperature 37 °C and pH 7.4 for 7 days. r_0 denotes the initial anisotropy.

System	r_0	$\langle \tau \rangle_{rot}^{\Psi}$ (ns)
Phase-separated β-LG	0.39	0.75
Dilute phase solution	0.37	0.85

$^{\Psi}$ = ±5%

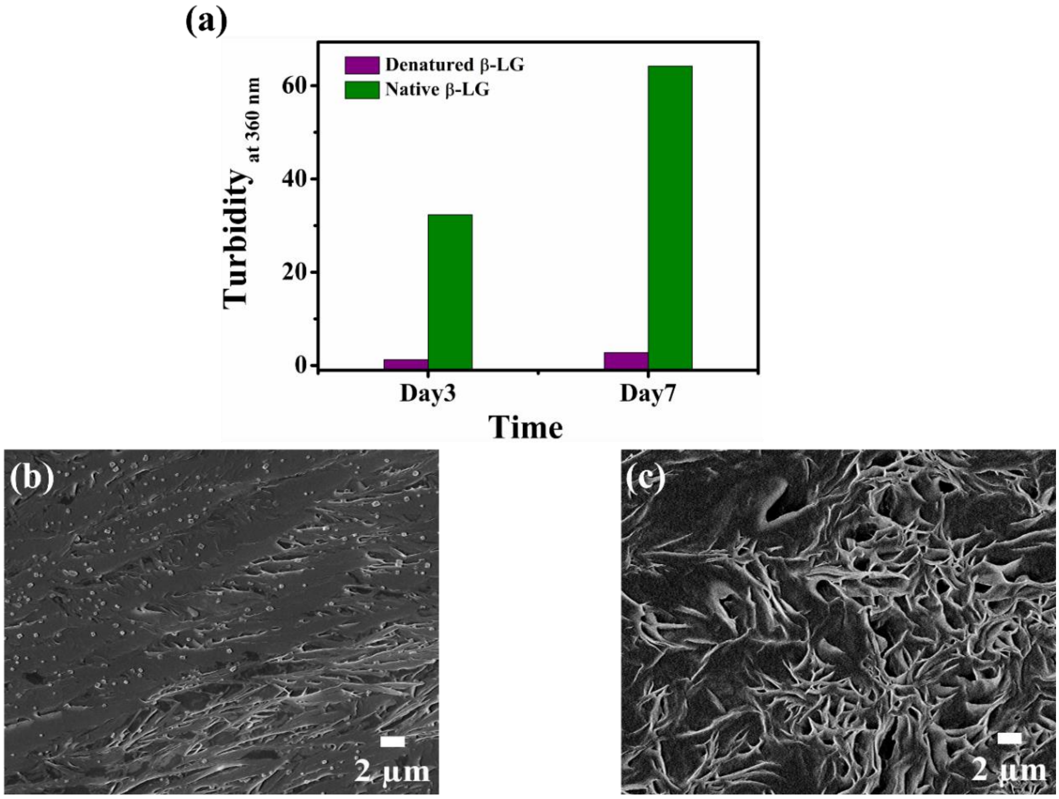

Fig. S9 Investigation of phase separation possibility with denatured protein. (a) Turbidity plot of the sample incubated with the native protein (sample contains 50 µM native β-LG incubated with 10% w/v PEG8000 at a temperature 37 °C and pH 7.4 after 7 days), and denatured protein (sample contains 50 µM of β-LG denature by GdHCl (6 M) incubated with 10% w/v PEG8000 at a temperature 37 °C and pH 7.4 after 7 days). (b) FESEM image of the GdHCl-denatured β-LG (6 M GdHCl, 50 µM β-LG) incubated in the presence of 10% w/v PEG8000 at a temperature 37 °C and pH 7.4 after 7 days. (c) FESEM image of the native β-LG sample incubated in the presence of 10% w/v PEG8000 at a temperature 37 °C and pH 2.3 after 7 days.

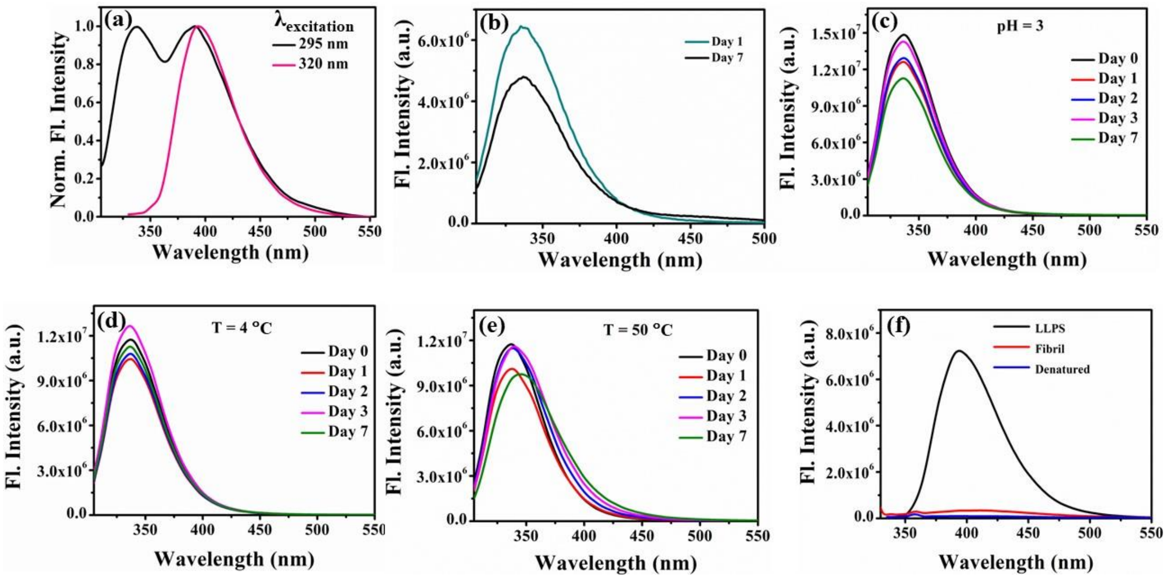

Fig. S10 (a) The CT band emission spectra on excitation at 295 nm and 320 nm. The fluorescence emission spectra of (b) β-LG without PEG8000 (c) β-LG incubated with PEG8000 at pH 3 when excited at 295 nm. (d) and (e) represent the fluorescence

emission spectra of β-LG incubated with PEG8000 at 4 °C and 50 °C, respectively when excited at 295 nm, indicating the absence of CT band (around 400 nm). (f) The comparative fluorescence emission spectra (λ_{ex} = 320 nm) of CT band emission of the native β-LG (undergoing LLPS, black) and denatured β-LG (not showing LLPS, blue) incubated with PEG8000 along with that of β-LG fibril.

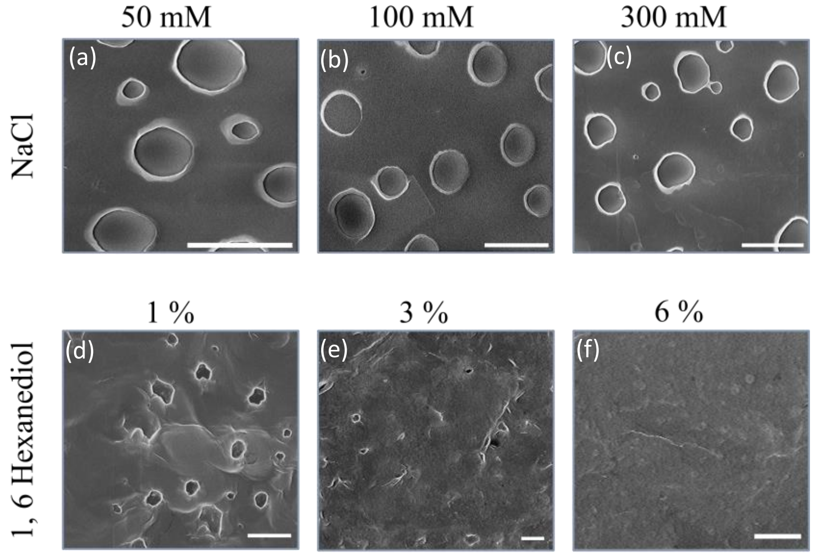

Fig. S11 The effect of electrolyte and aliphatic alcohol 1,6-hexanediol on the formation of droplets. (a), (b) and (c) represent the FESEM images of the sample incubated with increasing concentrations of NaCl (50-300 mM). (d), (e), and (f) represent the FESEM images of the sample incubated with increasing concentrations of 1, 6-hexanediol (1-6%) (scale bar: 5 μm).

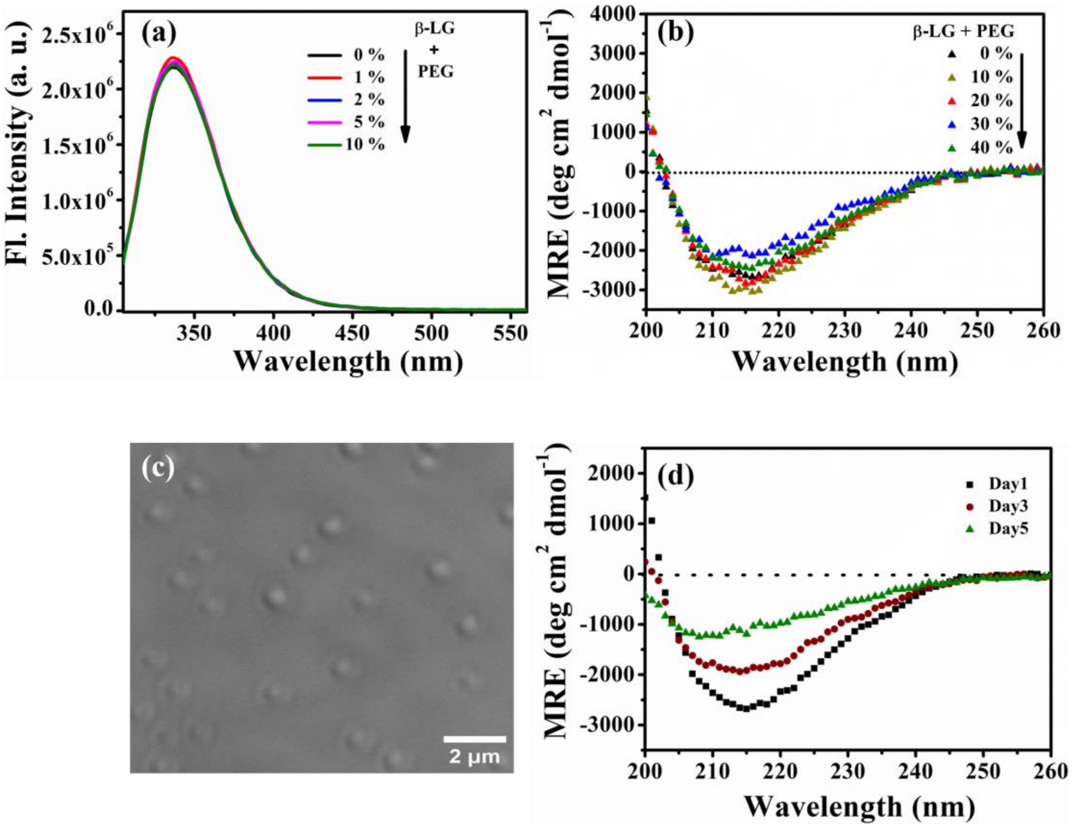

Fig. S12 Investigation of the role of PEG8000 on the photophysical and structural properties of the protein. (a) The fluorescence emission spectra of the intrinsic tryptophan fluorescence of the protein (50 μM) in the presence of varying concentration of PEG8000 (0-10% w/v) in the solution. (b) The CD spectra of the protein (5 μM) in the presence of varying concentration of PEG8000 (0-10% w/v). (c) FESEM images of the protein (50 μM) incubated with dextran (10% w/v), as an alternative inert synthetic polymer (crowder), at 37 °C and pH 7.4 after 7 days. (d) Day-wise CD spectra of the protein (50 μM) incubated with dextran (10% w/v) at 37 °C and pH 7.4.

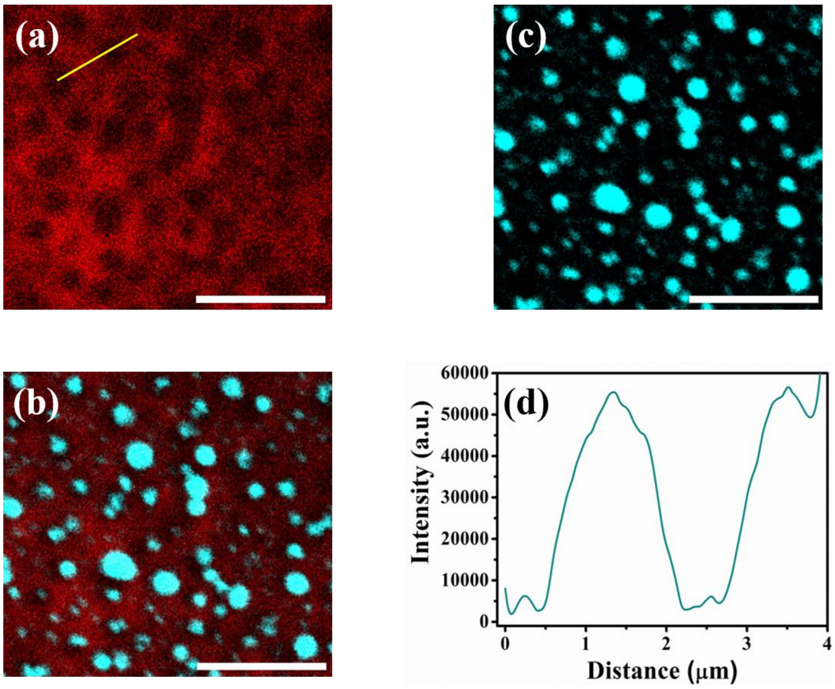

Fig. S13 Confocal fluorescence images of the β-LG condensates solution with 5-DTAF-labelled PEG8000. Panel (a) displays the monitoring through the FITC excitation and emission channel corresponding to 5-DTAF labelled to PEG8000 (condensate sample contained 1% labelled with 9% unlabelled PEG incubated with 50 μM β-LG at 37 °C, pH 7.5 for ~ 24 h; scale bar: 5 μm), while panel (b) represent monitoring through the DAPI excitation and emission channel corresponding to CPM-labelled β-LG (scale bar: 5 μm). Panel (c) demonstrates the colocalization of the images acquired from both the channels. The images are represented in false colour for better colour contrast (scale bar: 5 μm). Panel (d) illustrates the line intensity plot (obtained from Fig. S13a) measured across the droplets to depict the fluorescence of the PEG-labelled 5-DTAF substantiating the lack of intensity within the droplets.

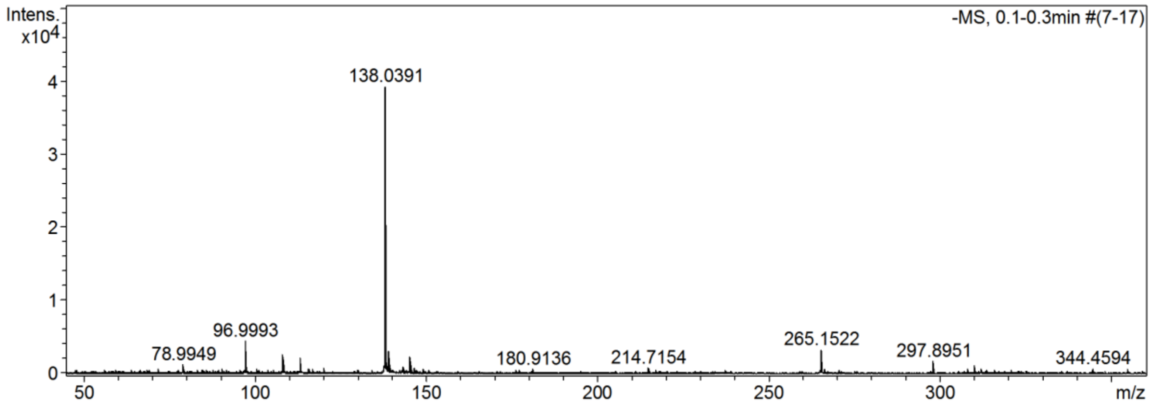

Fig. S14 ESI-MS spectra of the supernatant solution after hydrolysis of PNPA to PNP (obtained by centrifugation followed by filtration through syringe filter) showing m/z peak centered at 138.0391 corresponding to the phenolate (PNP) ion. The sample for centrifugation was obtained after hydrolysis of 120 μM PNPA by β-LG condensates (50 μM β-LG in 10% w/v PEG8000, at 37 °C and pH 7.4 after 7 days).

Table S4 Parameters (maximum velocity (V_{max}), Michaelis constant (K_m), and catalytic rate constant (k_{cat}) obtained from Michaelis-Menten enzymatic model for the catalytic activity towards the ester hydrolysis using β-LG condensates solution (50 µM β-LG in 10% w/v PEG8000, at 37 °C and pH 7.4 after 7 days), native β-LG, PEG8000 and PB.

**System**	**V_{max} (µM min^{-1})**	**K_m (µM)**	**k_{cat} (min^{-1}×10^3)**
PB	3.2 ± 0.2	1300 ± 100	64 ± 4
PEG	6 ± 1	4000 ± 800	120 ± 20
β-LG	7 ± 1	1200 ± 200	140 ± 20
LLPS	44 ± 1	900 ± 40	900 ± 20

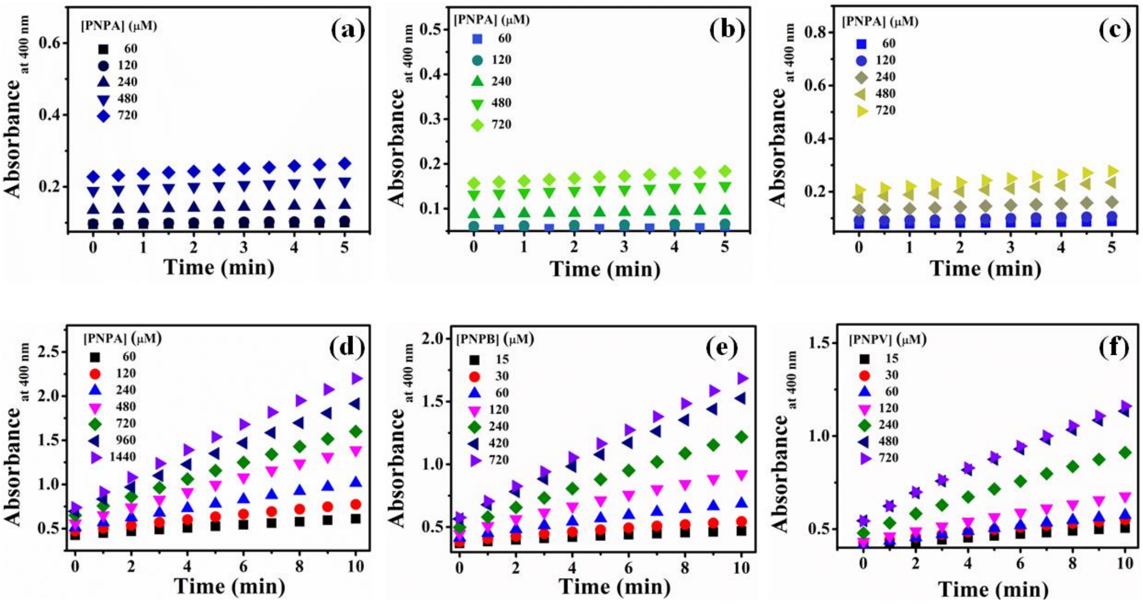

Fig. S15 Esterase kinetics of β-LG condensates (50 μM β-LG in 10% w/v PEG8000, at 37 ºC and pH 7.4 after 7 days) with varying concentrations of the PNPA (0-720 μM) for (a) PB, (b) PEG, (c) native β-LG. Esterase kinetics of phase-separated β-LG condensates (50 μM β-LG in 10% w/v PEG8000, at 37 ºC and pH 7.4 after 7 days) with varying concentrations (mentioned in the Figures) of the different substrates (d) PNPA, (e) PNPB, and (c) PNPV.

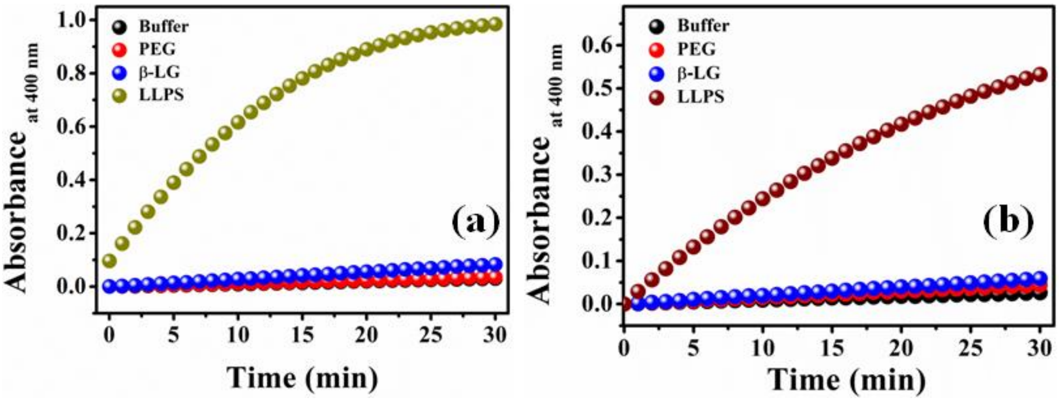

Fig. S16 Comparative esterase kinetics for β-LG condensates (50 μM β-LG in 10% w/v PEG8000, at 37 °C and pH 7.4 after 7 days) with PB, PEG8000, and native protein (β-LG) towards the ester substrates (a) PNPB and (b) PNPV.

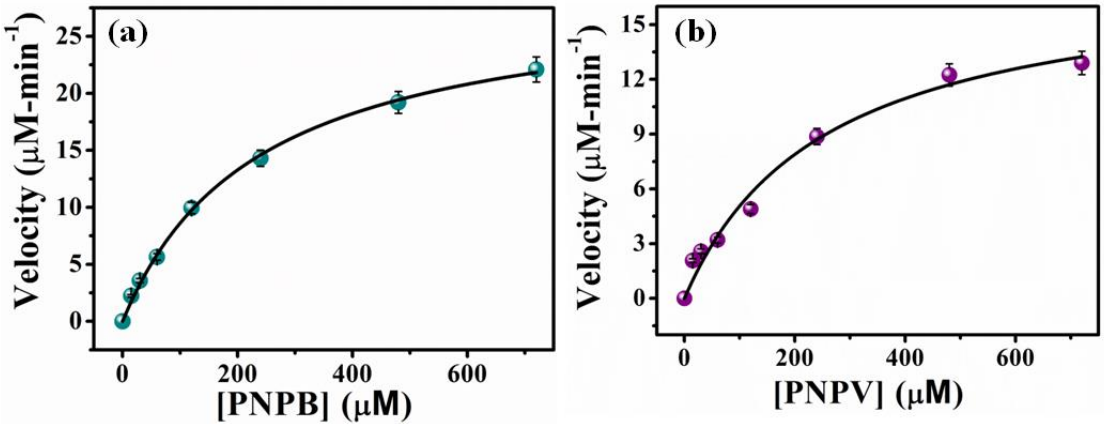

Fig. S17 Rate of change of velocity of the reaction with varying substrate concentration (15-720 μM) for (a) PNPB and (b) PNPV, fitted to the non-linear curve fitting for Michaelis-Menten enzyme kinetics model. Here the samples were investigated under varying concentrations of ester (PNPB and PNPV) substrates in the presence of β-LG condensates (50 μM β-LG in 10% w/v PEG8000, at 37 °C and pH 7.4 after 7 days).

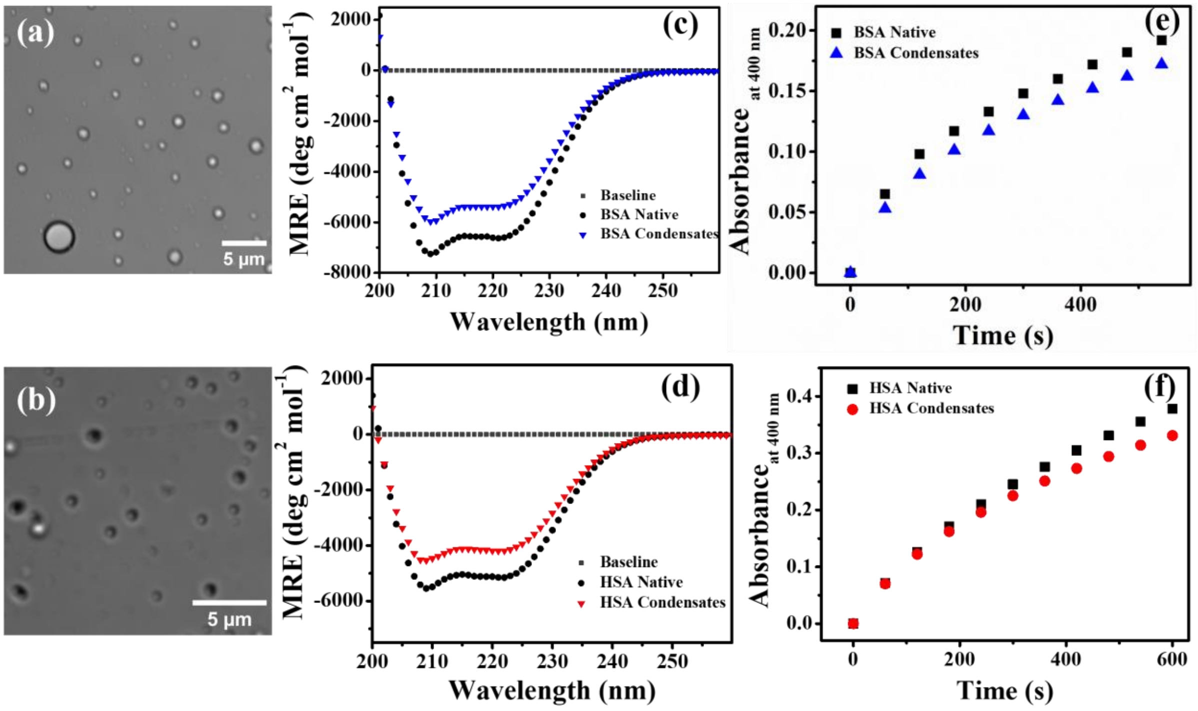

Fig. S18 Investigation of alteration in functional properties of other globular proteins (BSA and HSA) through esterase-like activity monitoring. (a) DIC image of the condensates of BSA (50 µM) when incubated with PEG8000 (10% w/v) at 37 °C and pH 7.4 for 7 days. (b) DIC images of the condensates of HSA (50 µM) when incubated with PEG8000 (10% w/v) at 37 °C and pH 7.4 for 7 days. (c) The CD spectra of the BSA condensates solution (50 µM BSA incubated with 10% w/v PEG8000 at 37 °C and pH 7.4 after 7 days; diluted to 5 µM). (d) The CD spectra of the HSA condensates solution (50 µM HSA incubated with 10% w/v PEG8000 at 37 °C and pH 7.4 after 7 days; diluted to 5 µM). (e) The esterase kinetics of the native and phase-separated BSA solution (50 µM BSA incubated with 10% w/v PEG8000 at 37 °C and pH 7.4 after 7 days). (f) The esterase kinetics of the native and phase-separated HSA solution (50 µM HSA incubated with 10% w/v PEG8000 at 37 °C and pH 7.4 after 7 days).

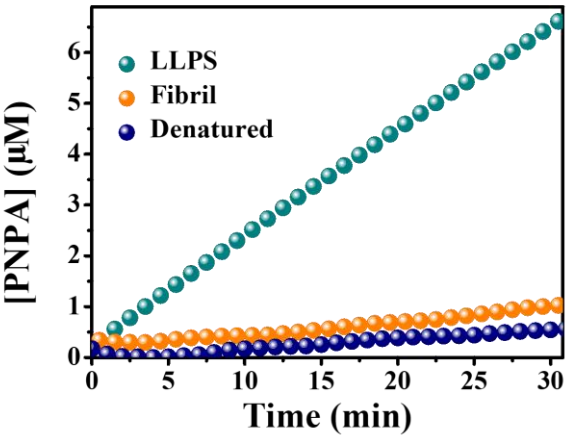

Fig. S19 The esterase kinetics of the LLPS system when formed through incubation with the native protein (50 µM) and the 6M GdHCl-denatured protein (50 µM) with PEG8000. The orange symbols represent the kinetic profile for the PNPA hydrolysis with the fibrillar aggregates form of protein in an equivalent concentration of β-LG (50 µM).

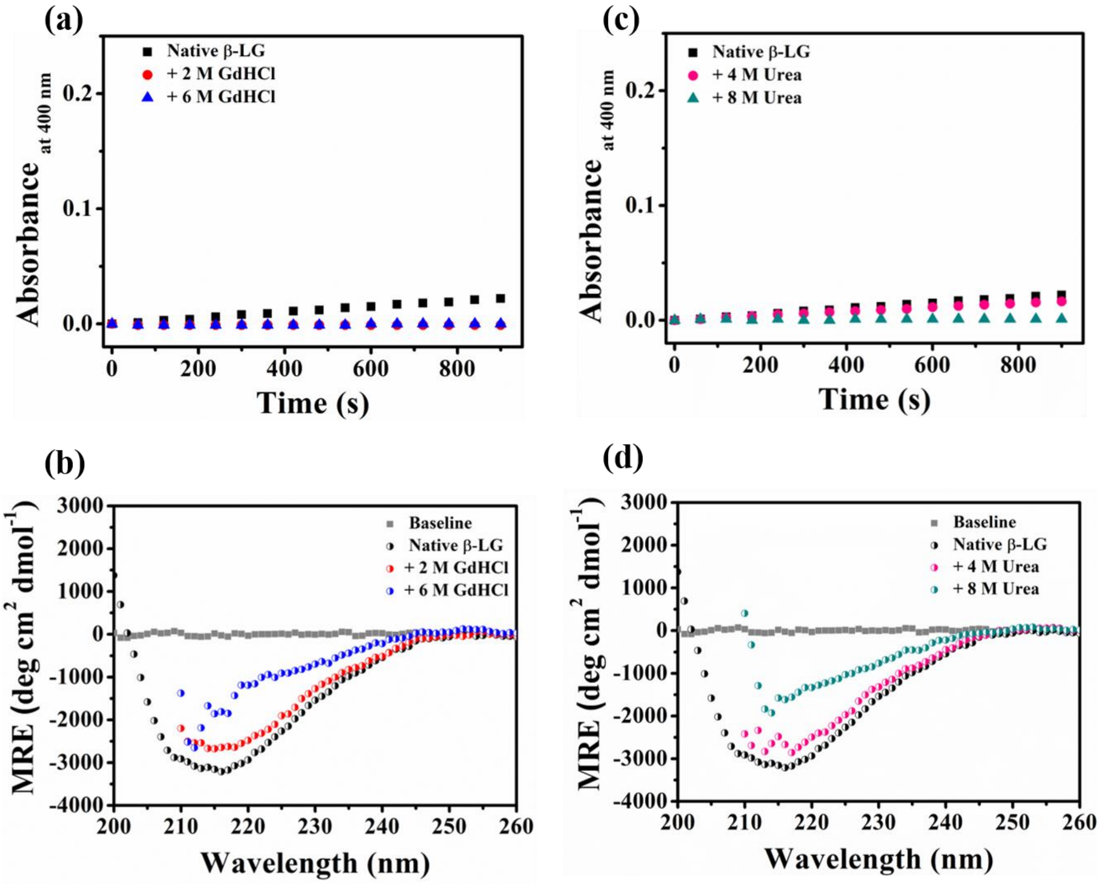

Fig. S20 (a) PNPA kinetics plots comparing the activity of native β-LG to that of the proteins denatured by 0-6 M GdHCl. (b) Illustrates the corresponding CD spectra for the native β-LG and the proteins denatured by 0-6 M GdHCl. (c) PNPA kinetics plots comparing the activity of native β-LG to that of the proteins denatured by 0-8 M Urea. (d) Illustrates the corresponding CD spectra for the native β-LG and the proteins denatured by 0-8 M Urea. It must be noted that the presence of denaturants (GdHCl and Urea) caused the HT value of the CD detector to shoot up beyond the permissible value for spectral acquisition below 210 nm. Hence, for the denatured protein, the spectra could not be recorded below 210 nm.

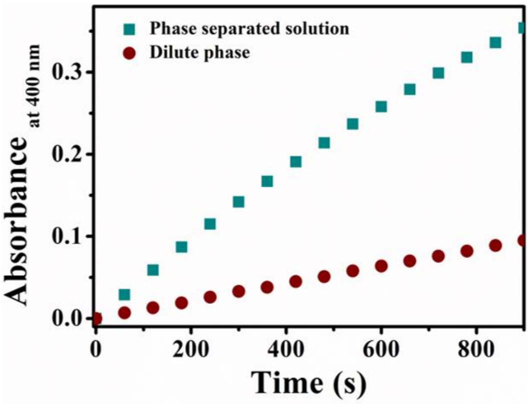

Fig. S21 Comparison plot of PNPA kinetics of the phase separated solution and dilute phase (after the centrifugation and separation of the condensed phase) using 120 μM PNPA.

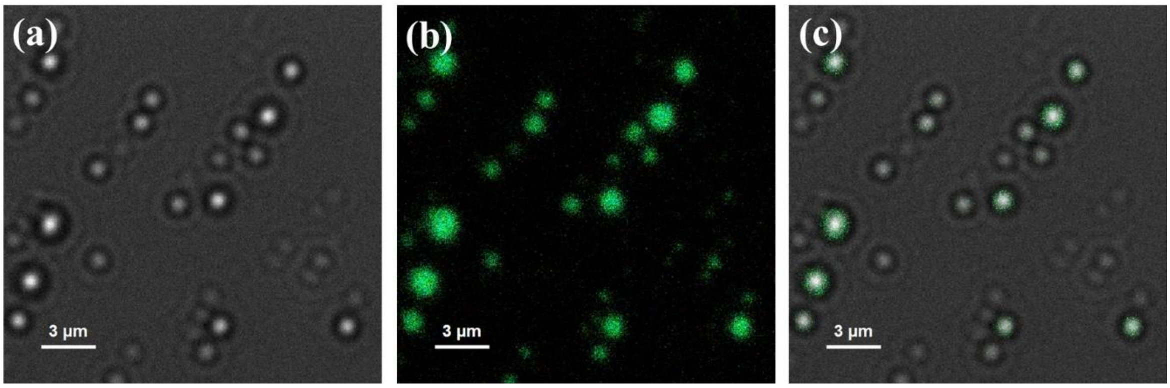

Fig. S22 The morphology of the condensates after catalysis and the droplet as a site of catalysis. (a), (b) and (c) respectively represent bright field, fluorescence and merge images of the droplets after the hydrolysis of the DCFDA to DCF using the condensates solution (where the protein β-LG concentration for droplet formation was 50 μM and the substrate, DCFDA concentration for hydrolysis was 10 μM; the images were recorded after 24 h of incubation).

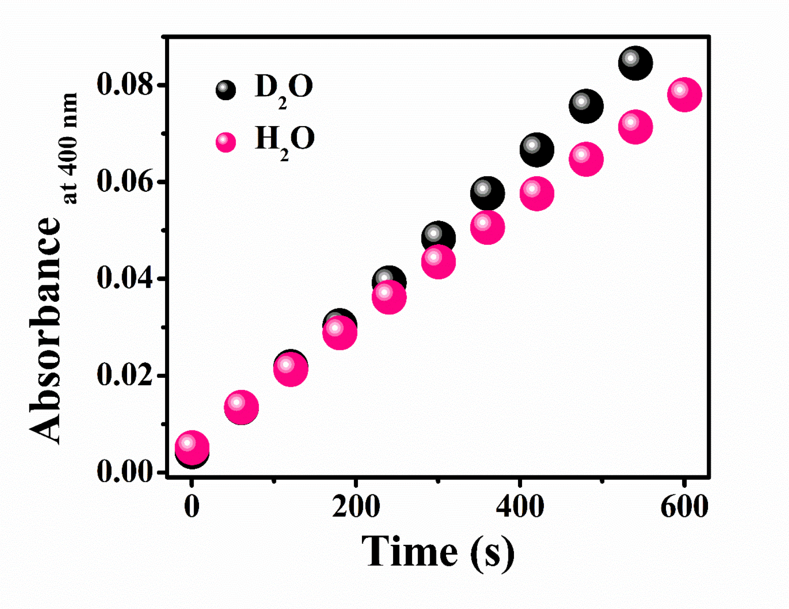

Fig. S23 Solvent isotopic effect on the catalytic profile. PNPA kinetics with phase-separated solution of β-LG half diluted with H_2O and D_2O (sample solution contains phase-separated solution of 50 µM β-LG in 10% w/v incubated at 37 °C after 5 days, ester substrate PNPA concentration of 120 µM).

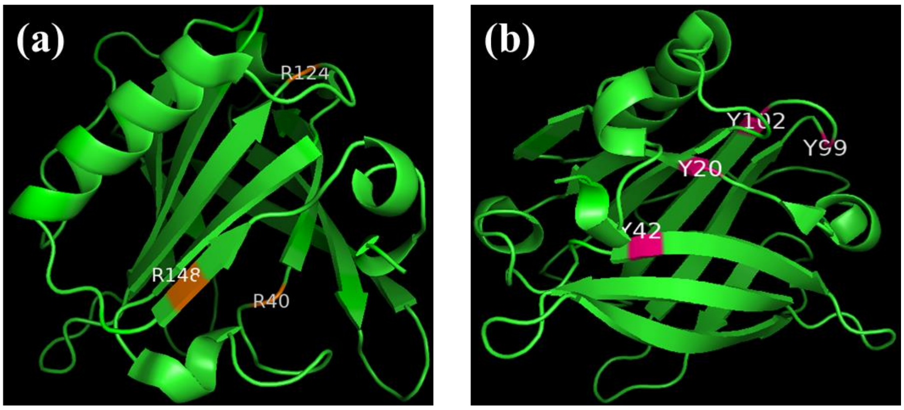

Fig. S24 Snapshot of the PDB structures of β-LG (analyzed through PyMol) showing the location of (a) arginine and (b) tyrosine residues present in β-LG.

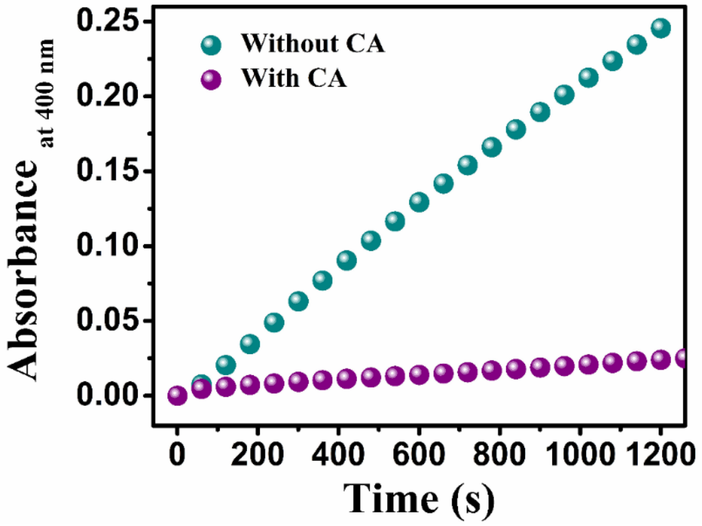

Fig. S25 Effect of caproic acid (CA) on the catalytic profile. PNPA kinetics with phase-separated solution of β-LG in the presence and absence of CA (sample solution contains phase-separated solution of 50 µM β-LG in 10% w/v PEG8000 incubated at 37 °C after 5 days, with ester substrate PNPA concentration of 120 µM; with or without CA, 0.0003 % w/v).

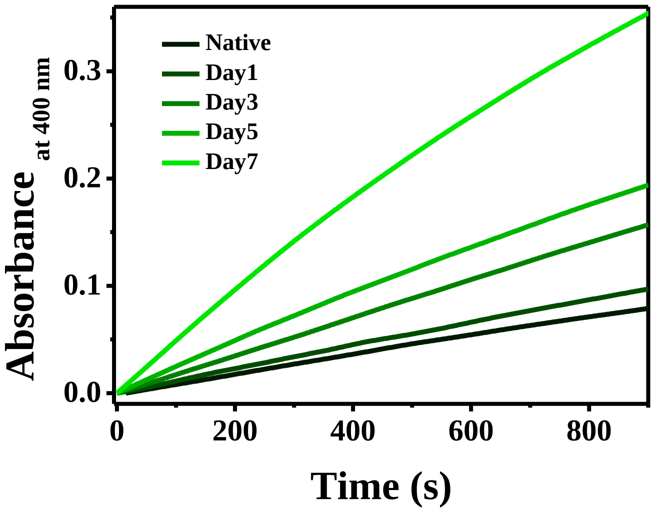

Fig. S26 The esterase kinetics of the phase separated system (50 µM β-LG incubated with 10% w/v PEG8000 at 37 °C) using PNPA (120 µM) as an ester substrate monitored at different times of incubation.

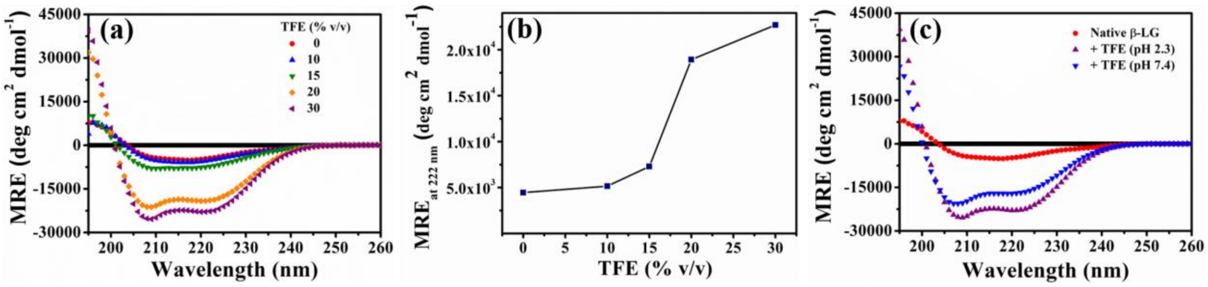

Fig. S27 (a) Far-UV CD spectra of β-LG (10 μM) under varying concentrations of TFE (0-30 % v/v) in aqueous HCl/KCl buffer at pH ~2.3 and 23 °C. (b) TFE concentration dependence of ellipticity at 222 nm for β-LG (10 μM) in aqueous HCl/KCl buffer at pH ~2.3 and 23 °C. (c) A comparative far-UV CD spectra of β-LG (10 μM) in the presence of 30 % v/v TFE in aqueous HCl/KCl buffer at pH ~2.3 and phosphate buffer at pH ~7.4 at 23 °C.

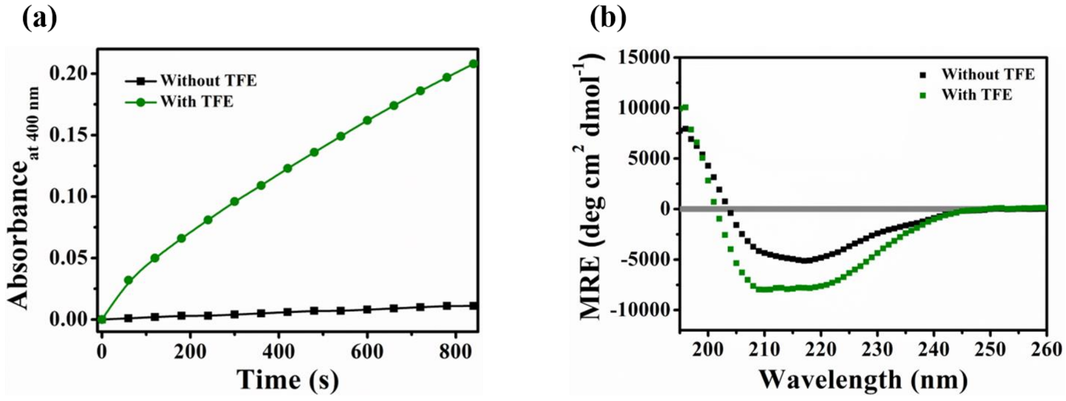

Fig. S28 (a) PNPA kinetics of β-LG (50 μM) in the presence (15% v/v) and absence (0% v/v) of TFE at 23 °C, pH 7.4 monitored by recording the absorbance at 400 nm. (b) A comparative far-UV CD spectra of β-LG (10 μM) in the presence (15% v/v) and absence (0% v/v) of TFE at 23 °C, pH 7.4.
